# CUL3-mediated NRF2 dysregulation in TP53-driven non-small cell lung cancer: neddylation-associated structural perturbations

**DOI:** 10.3389/fbinf.2026.1918128

**Published:** 2026-10-30

**Authors:** Poobana Dharmalingam, C. Sudandiradoss

**Affiliations:** Department of Biotechnology, School of Biosciences and Technology, Vellore Institute of Technology, Vellore, Tamil Nadu, India

**Keywords:** CRL3, cullin-3, EGFR, Fisher’s exact, neddylation, NRF2, NSCLC, TP53

## Abstract

CUL3 encodes Cullin-3, the scaffold of Cullin-RING Ligase 3 (CRL3), which mediates proteasomal degradation of NRF2 through KEAP1-dependent ubiquitination. While canonical NRF2 alterations such as KEAP1 or NFE2L2 mutations are well characterised in non-small cell lung cancer (NSCLC), the role of CUL3 mutations remains poorly defined. We hypothesised that CUL3 missense variants are conditionally enriched in TP53-altered NSCLC, with the EGFR-oncogenic subset as a defined disease background rather than a synergistic requirement. A multi-cohort NSCLC dataset (n = 47,862; 10,228 with oncogenic EGFR mutations and confirmed CUL3 coverage) was interrogated using two-layer Fisher’s exact testing and logistic regression with an EGFR × TP53 interaction term, and validated transcriptomically in TCGA LUAD/LUSC. CUL3 mutations showed conditional enrichment in the TP53-mutant EGFR background (OR = 2.92, p = 4.68 × 10^−29^); the non-significant EGFR × TP53 interaction (OR = 0.86, p = 0.547) confirmed that enrichment is driven by TP53 loss rather than EGFR synergy. CUL3-mutant tumours showed elevated NRF2 activation across three independent signatures, with increased proliferative signalling and altered drug metabolism. All-atom molecular dynamics simulations of wild-type, L321F, G402E and M711I CUL3:KEAP1 complexes in inactive and neddylation-active states revealed variant-specific conformational behaviour. L321F drove the neddylated complex into a conformationally restricted state with reduced backbone flexibility, decreased radius of gyration, and a shifted free energy minimum, whereas G402E showed progressive destabilisation and M711I remained comparable to wild-type. Free energy landscape analysis confirmed a shifted conformational energy minimum in L321F + NEDD8 relative to wild-type, indicating impaired catalytic flexibility despite intact complex formation. These findings define CUL3 as a TP53-associated modifier of NRF2 regulation within EGFR-driven NSCLC, operating through allosteric rigidification of the neddylation-active CRL3 complex.

## 1 Introduction

Carcinogenesis is shaped by the accumulation of genetic alterations, but the functional impact of these alterations depends not only on their individual roles but also on the broader molecular environment in which they occur (Laconi et al., 2020). Over the past few years, cancer genomics has changed the paradigm of single-gene drivers to combinatoric patterns of mutation, with interactions between co-occurring alterations influencing tumor behaviour, evolution and response to therapy (Sinkala, 2023). Regardless of the developments, numerous low-frequency mutations have not been well understood, pertaining to whether they have any meaningful contribution to the tumor biology or just represent a background mutational process (Brown et al., 2019).

Non-small cell lung cancer (NSCLC) represents a prototypical example of a genomically stratified disease (Robertson et al., 2020). Activating mutations in EGFR define a major subset of NSCLC characterized by dependence on receptor tyrosine kinase signaling and sensitivity to targeted therapies (Sun et al., 2022). However, EGFR-oncogenic tumors are not biologically uniform. Comorbid variants, especially in TP53, are common and have been associated with increased genomic instability, distortions in stress responses, and variability in clinical outcomes (Canale et al., 2022). These findings indicate that EGFR-mediated oncogenesis is regulated by further levels of genomic context, resulting in discrete biological subgroups among which is typically considered a single molecular cluster.

In this context, identifying other alterations that operate within the *EGFR* or *EGFR-TP53* background is also a crucial but underexplored area. One of them is Cullin 3 (*CUL3*), which is a core scaffold protein of Cullin-RING E3 ubiquitin ligase complexes (Genschik et al., 2013). These complexes control the processes of protein turnover via ubiquitination and are the key regulators of cellular homeostasis (Cui et al., 2016). One of the most important regulatory characteristics of Cullin-based ligases is that they depend on the neddylation, which involves the conjugation of NEDD8 and activation of ligase complex allowing the recognition of substrates (Chiba and Tanaka, 2004). In this way, *CUL3* is involved in a variety of cellular mechanisms, such as cell cycle regulation, proteostasis, and environmental stress adaptive responses (Chen and Chen, 2016).

Among the most described functions of CUL3 is its involvement in the regulation of the KEAP1-NRF2 pathway (Taguchi et al., 2011). In normal conditions, CUL3 is complexed with KEAP1 to mediate ubiquitination and degradation of NRF2, a transcription factor that regulates the expression of antioxidant and cytoprotective genes (Cullinan et al., 2004). Disruption of this regulatory axis causes stabilization of NRF2 and the programs of oxidative stress responses, able to support tumor survival in the conditions of metabolic and therapeutic stress (Barrera et al., 2021). The changes in KEAP1 and NFE2L2 (protein that codes NRF2) in NSCLC have been extensively documented and associated with aggressive tumor phenotype and resistance to therapy (Singh et al., 2021; Xu et al., 2023).

The molecular logic by which the CUL3–KEAP1 axis controls NRF2 including the roles of neddylation and deneddylation in cycling CRL3 activity has been reviewed in detail (Baird and Yamamoto, 2020). That body of work establishes the canonical, wild-type regulatory mechanism. The present study is distinct in three respects. First, rather than describing the normal pathway, it asks whether disease-associated CUL3 missense variants are non-randomly distributed with respect to genomic context in NSCLC, and identifies a specific TP53-defined enrichment. Second, it links CUL3-variant status to a functional transcriptional readout by coupling NRF2 pathway activation to neddylation-machinery co-expression in patient tumors. Third, it provides variant-resolved structural and molecular-dynamics evidence for how a specific substitution perturbs the neddylation-active CRL3 conformation. The contribution therefore lies not in re-establishing that CUL3 regulates NRF2, but in defining the genomic context, functional correlates, and structural consequences of CUL3 variation in NSCLC.

Importantly, the NRF2-mediated oxidative stress response represents a survival mechanism that is distinct from classical apoptotic pathways (Kapuy et al., 2018). In the context of EGFR-oncogenic NSCLC, co-occurring TP53 alterations compromise apoptotic control, whereas additional perturbations in CUL3-mediated regulation may shift tumor cells toward oxidative stress–adaptive survival mechanisms (Guo et al., 2020; Canale et al., 2022). This distinction highlights the potential importance of regulatory components such as CUL3, which influence how cells respond to stress rather than directly inducing cell death. Therefore, the role of CUL3 itself in the biology of NSCLC remains unclear despite its key role in this regulatory network (Hellyer et al., 2019). Recent clinical data has also started to indicate the possible clinical relevance of this rare genomic configuration. There was a reported EGFR/TP53/CUL3 triple-mutant NSCLC case with a rapid disease progression and widespread metastases, and is likely to respond poorly to standard EGFR-targeting therapy (Osimertinib) with a highly shortened median overall survival rate (32.6 months for EGFR/TP53/CUL3) compared to the EGFR-only (84 months) and EGFR/TP53 (42.2 months) groups (Hatano et al., 2025). Despite these observations, the mechanistic basis of this aggressive phenotype has not been fully explored.

Unlike KEAP1 or NFE2L2, mutations in CUL3 are relatively infrequent, and their significance has not been systematically characterized (Robertson et al., 2020). Existing studies have largely focused on canonical NRF2-axis alterations, leaving open the question of whether CUL3 mutations represent independent biological events or simply reflect perturbations within the same pathway (Hu et al., 2012; Dempke and Reck, 2021; Kinslow et al., 2022). In addition, it remains unknown whether CUL3 changes are randomly distributed in tumors or preferentially linked to particular oncogenic settings, e.g., EGFR-driven or EGFR-TP53 co-mutant environments. The answer to this question is necessary to define the role of CUL3 as a passive component of tumor evolution or a conditional modifier with functional relevance.

A significant challenge in the understanding of such low-frequency mutations is their limitation in differentiation of meaningful patterns and random noise (Erdem-Eraslan et al., 2015). Conventional mutation frequency analysis can overlook structured interactions that can only be identified when mutations are studied with respect to particular genomic backgrounds. Here, a coarse-grained method of analysis that focuses on the patterns of co-occurrence and interaction rather than isolated mutation can serve as a useful approach to detecting context-specific signals in large genomic data, and can be generalised to structural modeling of protein complexes to determine the functional implications of such changes. Characterising the structural consequences of such variants through experimental approaches is also a considerable challenge, as the neddylation-active conformation of the CRL3 complex is transient and not readily amenable to standard biochemical or crystallographic resolution, and in this regard, computational structural modelling and all-atom molecular dynamics simulation can serve as a tractable and mechanistically informative approach to determine how specific CUL3 missense variants alter the conformational geometry of the active E3 ligase complex.

In this study, we hypothesize that CUL3 mutations are not randomly distributed in NSCLC but instead exhibit context-dependent enrichment within specific oncogenic backgrounds, particularly within TP53-altered tumors of which EGFR-oncogenic NSCLC represents one clinically defined subset rather than requiring the combined presence of EGFR and TP53 alterations. We also propose that this enrichment may represent a distinct biological condition associated with altered stress-response regulation, rather than a simple reflection of canonical NRF2-axis changes. To test this hypothesis, we conducted a multi-cohort genomic analysis of NSCLC combining co-mutation patterns, statistical interaction modelling, and pathway-level transcriptomic interpretation, followed by all-atom molecular dynamics simulation of the CUL3:KEAP1 complex in wild-type and L321F mutant forms across both inactive and neddylation-active states. Taken together, these analyses are expected to define CUL3 as a context-dependent modifier of NRF2 regulation in this subgroup, provide mechanistic insight into how CUL3 perturbation contributes to stress-response dysregulation in TP53-altered tumors, and establish a structural basis relevant to understanding how CUL3 variants may perturb the neddylation-active CRL3 complex within this genomic context.

## 2 Materials and methods

### 2.1 Data sources and cohort assembly

The Cancer Genome Atlas (TCGA), MSK-IMPACT, AACR GENIE v19.0, and CPTAC were employed in accessing somatic mutation and clinical data on non-small cell lung cancer (NSCLC) (Cheng et al., 2015; Sweeney et al., 2017). All the datasets were retrieved in cBioPortal or corresponding consortium portals (Cerami et al., 2012; Gao et al., 2013). The samples that had histologically confirmed NSCLC (lung adenocarcinoma, squamous cell carcinoma, or unspecified NSCLC) and had mutation data were only included. To avoid duplication across overlapping repositories, patient-level deduplication was performed using sample identifiers and cohort priority rules (TCGA > MSK-IMPACT > CPTAC > GENIE > others). The final pooled dataset consisted of 47,862 distinct NSCLC samples. Primary analyses used AACR GENIE v19.0; the subsequently released AACR GENIE v20.0 was incorporated at the revision stage solely as an independent large-cohort validation of the driver-context and histology analyses, and does not substitute for any primary result. Due to the differences in sequencing panel between cohorts, downstream filtering took into explicit account gene coverage to avoid the possibility that absence of mutation call was due to panel limitations.

### 2.2 Mutation filtering and frequency mapping

The analysis was limited to patients harbouring oncogenic EGFR mutations. These were canonical activating mutations, which were exon 19 deletions, L858R, T790M, exon 20 insertions and annotated activating variants. Mutation data have been filtered to retain only non-silent variants, including nonsense, missense, frameshift, splice-site mutations. Synonymous variants were excluded. Samples were retained only if sequencing panel comprised coverage for CUL3 to ensure accurate classification of mutation status. Hypermutator samples were defined using the MSK-IMPACT criterion of tumor mutational burden ≥100 mutations/Mb and were excluded from the canonical triple-positive cohort to remove the confounding effect of mutational instability on co-occurrence statistics.

In the EGFR-oncogenic category, patients were stratified into three groups according to the co-mutation status; EGFR-only, EGFR + TP53 and EGFR + TP53+CUL3. The canonical Triple-Positive cohort was further refined by exclusion of NFE2L2-mutant and hypermutator samples to remove patients with independent NRF2 gain-of-function or genomic-instability confounders. This stratification allowed the assessment of CUL3 mutations in a specific genomic background and not in heterogeneous cases of NSCLC. The frequencies of co-mutation were computed among all cohorts and visualised with proportional bar graphs and mutation rate distributions. In order to evaluate the reproducibility in the datasets of different sequencing depths, the rates of CUL3 mutation were calculated in each cohort separately. Such analyses also stated that the observed patterns of mutation were not different in independent sources and not due to technical artefacts.

### 2.3 Fisher’s exact test and interaction modeling

Association testing was conducted through Fisher’s exact test and logistic regression modeling to evaluate the occurrence of CUL3 mutations in certain genomic contexts (Lury and Fisher, 1972). The exact test through Fisher was used at two levels: on the entire NSCLC cohort and on the EGFR-oncogenic subset. Odds ratios (OR) and 95% confidence intervals were computed, and p-values were adjusted with the Benjamini–Hochberg false discovery rate (FDR) correction (Benjamini and Hochberg, 1995).

A multivariate logistic regression model was developed to evaluate non-additive interactions, in which CUL3 mutation status is the dependent variable, and the predictors are EGFR mutation, TP53 mutation, and EGFR mutation x TP53 mutation. This model allowed the independent effects and any context-dependent interaction between EGFR and TP53 on CUL3 enrichment to be quantified. All statistical computations were performed in R (version 4.3.1) and Python, with figures generated in R using ggplot2 and ComplexHeatmap.

### 2.4 NRF2-axis confound control and genomic severity proxies

To determine whether observed CUL3-associated patterns could be explained by canonical NRF2 pathway alterations, co-mutation rates of KEAP1, NFE2L2, and STK11 were evaluated within the EGFR + TP53 + CUL3 group. A pre-defined threshold of 15% co-mutation frequency was used to identify potential confounders requiring sensitivity analysis.

A series of six exclusion scenarios were applied, which sequentially eliminated samples with mutations in the NRF2-axis to test the robustness of the observed patterns. Considering that there is no equally reliable survival data for all the cohorts, the genomic severity was determined by other proxies. Tumor mutational burden (TMB) was calculated as mutations per megabase and the mutation groups were compared by using the Mann-Whitney U test with FDR correction (Mann and Whitney, 1947). Additionally, biopsy origin (primary vs. metastatic) was used as a contextual indicator of disease stage where available. These measures provided consistent cross-cohort indicators of tumor aggressiveness without relying on incomplete survival datasets.

### 2.5 Transcriptomic cohort definition and NRF2 pathway activity scoring

Transcriptomic analysis was performed using RNA-sequencing data from The Cancer Genome Atlas (TCGA) lung adenocarcinoma (LUAD) and lung squamous cell carcinoma (LUSC) cohorts, as these represent the only datasets within the assembled multi-cohort framework with matched transcriptomic profiles. Raw expression data were obtained as Z-score–normalized RSEM values relative to the pan-cancer reference distribution.

To ensure consistency with the genomic filtering strategy, a sequential eligibility framework was applied. Patients harbouring activating NFE2L2 mutations or hypermutator status (tumor mutational burden ≥100 mutations/Mb) were excluded to avoid confounding NRF2 pathway activation. EGFR-oncogenic patients found in the genomic dataset were limited to the wild-type reference group, whereas the rest of the samples with CUL3-variants were held based on direct mutational annotation regardless of cohort overlap. The last transcriptomic cohort was 120-patient, comprising 89 wild-type and 29 CUL3-mutant, and two patients of the canonical triple-positive subgroup.

NRF2 pathway activation was quantified using multiple independently validated gene signatures to ensure robustness and avoid circular inference. These included the Hallmark_Reactive_Oxygen_Species pathway from MSigDB, along with curated NRF2 target gene sets described by Singh et al. and Best et al. Pathway activity was computed as an arithmetic mean of Z-score normalised expression values of all genes in each signature, per sample (Singh et al., 2008; Best et al., 2018). This method was chosen instead of enrichment-based methods because NRF2 gene sets are relatively small, and one requires stable per-sample estimates. The genes that were not in the expression matrix were not computed. Two-sided Wilcoxon rank-sum tests with the correction of multiple testing with Benjamini–Hochberg were used to conduct statistical comparisons between wild-type and CUL3-altered groups.

### 2.6 Immune microenvironment profiling

To characterize the tumor immune landscape, single-sample gene set enrichment analysis (ssGSEA) was applied to log_2_-transformed expression values using the GSVA framework (Hänzelmann et al., 2013). Immune signatures representing major cell types and biological processes, including B cells, T cells, NK cells, macrophages, monocytes, neutrophils, plasma cells, and proliferation, were derived from the imSig reference panel.

Each sample had signature scores calculated and Wilcoxon rank-sum tests with a false discovery rate correction were used to determine the difference detected in the groups of wild-type and CUL3-altered tumors. Since limited number of triple-positive samples were observed, these were represented individually rather than aggregated.

### 2.7 NRF2-regulated drug metabolism gene analysis

A targeted panel of NRF2-controlled genes of drug metabolism were analysed to determine the possible functional outcomes of pathway activation. This panel was composed of Phase I enzymes (CYP family), Phase II conjugation enzymes (UGT family), ATP-binding cassettes transporters, and glutathione transferases which were chosen on the basis of known NRF2 transcriptional regulation and importance to therapeutic metabolism.

The difference in expression between the groups were measured by comparing the median Z-scores and the statistical significance was determined by the Wilcoxon rank-sum analysis with multiple corrections (Wilcoxon, 1945). To acquire normalized expression values, the differences were described as ΔZ (difference in median Z-scores) instead of fold change.

### 2.8 Neddylation pathway co-expression analysis

In order to examine the connection between neddylation machinery and NRF2 activation, the expression profiles of the main components of the pathway were determined, such as NAE1, UBA3, UBE2M, UBE2F, RBX1, SENP8, KEAP1, CUL3, and NFE2L2.

Group-level expression differences were measured by Wilcoxon rank-sum tests, whereas Spearman rank correlation analysis was used in measuring the association between gene expression and NRF2 activity (Spearman, 1904). This kind of dual approach enabled the determination of the differential expression and coordinated pathway activity. The statistical tests were all corrected for multiple comparisons with the Benjamini–Hochberg procedure (Benjamini and Hochberg, 1995).

### 2.9 Structural modeling of the CUL3–KEAP1 complex

To investigate the structural consequences of CUL3 perturbation within the defined genomic context, a reduced structural system comprising CUL3 and KEAP1 was constructed. EGFR, TP53, and CUL3 were not modelled as a single complex, as these proteins operate in distinct cellular compartments and do not form a direct physical assembly; their co-occurrence reflects a systems-level genomic context rather than a structural interaction.

A crystal structure of PDB 5NLB was used as a template of the KEAP1 BTB dimer and interaction with the CUL3 N-terminal region to generate the wild-type CUL3 N-terminal-KEAP1 complex. The full-length of CUL3 structure was modelled in Neurosnap platform with Alphafold2 and structurally aligned with the template that was bound to KEAP1 to maintain the experimentally resolved interface (Jumper et al., 2021; Boitreaud et al., 2024). A neddylation-compatible CUL3 conformation was also independently produced and served as the reference scaffold, onto which KEAP1 was repositioned by structural alignment of the CUL3 backbone using pymol (Delano, 2002). In this way, it was possible to reconstruct a unified CUL3:KEAP1 system and maintain both the integrity of the interface and the compatibility with the active Cullin conformation. The modelled complex, the NEDD8 linkage and the positions of the modelled variant sites are presented in Section 3.11.

Candidate CUL3 missense variants were prioritised using a composite scheme combining (i) occurrence in the assembled cohorts, (ii) localisation within structurally interpretable segments of the cullin scaffold of the canonical CUL3 isoform (ENST00000264414; 768 aa), (iii) predicted functional impact from complementary *in silico* predictors, and (iv) mechanistic suitability for modelling the neddylation-active CRL3 state. In-silico functional prediction tools such as SIFT and PolyPhen-2 were also used to determine the potential effect of the candidate mutations to support prioritisation of variants (Ng and Henikoff, 2003; Adzhubei et al., 2010). The resulting variants were then introduced into the structural model in SwissPBD viewer with the proper optimization of the side-chains and subsequent energy minimization carried using yasara energy minimization interface to generate mutant complex (Guex, N. and Peitsch, 1997; Krieger et al., 2009). All the generated structures were validated by analysing their Ramachandran scores in Ramplot (Kumar and Rathore, 2025).

### 2.10 Neddylation modelling and isopeptide bond construction

Activation of CRL3 ligase activity requires covalent conjugation of NEDD8 to CUL3 K712 through an isopeptide bond between the K712 ε-amino group (NZ atom) and the C-terminal carboxyl group of NEDD8 G76. As this covalent linkage is absent from available crystallographic structures of cullin–NEDD8 complexes, it was constructed manually for the neddylation-active simulation systems. Atom typing for the bond-forming residues was modified to reflect the formed amide chemistry: K712 NZ was reassigned from atom type NH3 to NH1 with one ε-hydrogen reassigned from HC to H, and NEDD8 G76 carbonyl carbon was reassigned from CC to C, with partial charges redistributed to maintain charge neutrality across the linkage region. Because the GROMACS (intermolecular_interactions) directive provides limited stability for cross-chain bonded parameters, the CUL3 and NEDD8 chains were merged into a single topology unit, allowing the new bond, angles, dihedrals, impropers, and 1–4 pair interactions to be defined within standard topology sections. Any steric clashes detected during initial geometry construction was resolved by side-chain rotamer adjustment followed by short steepest-descent minimisation. The same construction protocol was applied identically to the wild-type and L321F mutant systems.

### 2.11 Molecular dynamics simulation and system preparation

To investigate the conformational dynamics of the CUL3:KEAP1 system, all-atom molecular dynamics simulations were performed for both wild-type and mutant structures in their non-neddylated and neddylated states. All simulations were carried out using GROMACS with the CHARMM36m all-atom force field (Bekker et al., 1993; Abraham et al., 2015). Protein topologies were generated following structure preparation, including incorporation of the covalent linkage for the neddylated systems. Each system was solvated in a cubic box with explicit water molecules and neutralized by addition of counter-ions, followed by adjustment to physiological ionic strength.

Energy minimization was first performed using the steepest descent algorithm to remove steric clashes and optimize initial geometry. This was followed by a two-stage equilibration protocol under periodic boundary conditions, consisting of constant volume (NVT) and constant pressure (NPT) ensembles with positional restraints applied to heavy atoms. Temperature was maintained at 310 K using a velocity-rescaling thermostat, and pressure was controlled at 1 bar using a Parrinello–Rahman barostat. Then, each system was run for a production MD of 200ns.

The analyses of the trajectories were conducted to define structural stability and dynamic behaviour, encompassing root mean square deviation (RMSD), root mean square fluctuation (RMSF), radius of gyration, solvent-accessible surface area (SASA), hydrogen bonding profiles, and principal component analysis with free energy landscape construction using gmx covar, gmx anaeig, and gmx sham. All plots were generated using Python (matplotlib and scipy libraries).

## 3 Results and discussion

### 3.1 Multi-cohort landscape of CUL3 mutations in EGFR-driven NSCLC

A total of 47,862 NSCLC samples were analysed across TCGA, MSK-IMPACT, CPTAC, and AACR GENIE datasets (Table 1). Within this pool, 10,228 samples harboured oncogenic EGFR mutations with confirmed CUL3 coverage (Supplementary Figure S1). Among these, 100 cases exhibited CUL3 mutations, and 76 samples were identified as triple-positive (EGFR + TP53 + CUL3) prior to confound exclusion. After sequential exclusion of hypermutators and NFE2L2-mutant tumors, the canonical Triple-Positive cohort comprised 61 patients.

**TABLE 1:** Multi-cohort sample composition and frequency of CUL3 mutations in EGFR-oncogenic non-small cell lung cancer. Patient-level deduplicated counts across five independent NSCLC sequencing repositories integrated through cBioPortal, summarising the number of EGFR-oncogenic, CUL3-mutant, and triple-positive (EGFR + TP53 + CUL3) samples and the per-cohort CUL3 mutation rate within the EGFR-oncogenic background.

Data source	Total samples	EGFR-oncogenic	CUL3-mutant	Triple-positive (EGFR/TP53 /CUL3)	CUL3 rate in EGFR background (%)
TCGA	3,279	291	5	5	1.72
MSK-IMPACT	5,068	1,593	8	4	0.5
AACR-GENIE (v19)	36,673	7,891	84	51	1.06
CPTAC	566	119	2	0	1.68
OTHER	2,276	334	1	1	0.3
Pooled (deduplicated)	47,862	10,228	100	61	0.98

Triple-positive count corresponds to the canonical N = 61 cohort retained after exclusion of NFE2L2-mutant and hypermutator (TMB ≥100 mut/Mb) samples; pre-sensitivity count was N = 76. CUL3 mutations are non-silent missense, nonsense, or frameshift variants. The pooled rate is computed as 100/10,228 = 0.98%. AACR-GENIE v19 contributed the majority of triple-positive samples (51/61, 83.6%).

Per-cohort CUL3 mutation rates within the EGFR-oncogenic background ranged from 0.30% (OTHER, 1/334) and 0.50% (MSK-IMPACT, 8/1,593) to 1.06% (AACR-GENIE, 84/7,891), 1.68% (CPTAC, 2/119), and 1.72% (TCGA, 5/291), with a pooled rate of 0.98% (100/10,228) (Table 1). The reduced rate observed in MSK-IMPACT is consistent with its targeted-panel design relative to the exome-based TCGA and CPTAC platforms. Despite this technical variation, the consistent rarity of CUL3 mutations across independent cohorts argues against a systematic sequencing or panel artefact and supports a stable biological frequency in EGFR-oncogenic NSCLC.

Cross-cohort consistency is particularly important given the heterogeneity of sequencing platforms and patient populations represented in these datasets. CUL3 mutations were detected across all five cohorts, and triple-positive cases were identified in four of five (TCGA, MSK-IMPACT, AACR-GENIE, and OTHER); their absence in CPTAC reflects the small EGFR-oncogenic denominator (n = 119) of that cohort rather than a true absence. The stable, low-frequency occurrence of triple-positive samples across independent populations argues against dataset-specific artefact and justifies further context-specific investigation rather than dismissing CUL3 as an incidental alteration.

### 3.2 Context-dependent enrichment of CUL3 mutations

Association testing demonstrated that CUL3 mutations are significantly allied with TP53 mutations both in the full NSCLC cohort and within the EGFR-oncogenic subset. In the full cohort (Layer 1; n = 24,120), TP53–CUL3 co-occurrence yielded an odds ratio (OR) of 2.81 (95% CI 2.36–3.36; FDR-adjusted p = 4.08 × 10^−35^), while in the EGFR-oncogenic subset (Layer 2; n = 10,228, of which 5,727 were TP53-mutant), the OR remained significant at 2.51 (95% CI 1.56–4.16; FDR = 4.14 × 10^−5^) (Table 2). These values demonstrate that CUL3 mutations co-occur with TP53 mutations more frequently than expected by chance, both at the population level and within the EGFR-oncogenic background. The concordance between the two analytical layers indicates a non-random co-mutational pattern that is preserved when the analysis is restricted to a single oncogenic context, motivating a formal interaction-modelling test to determine whether this enrichment reflects TP53 main effect, EGFR main effect, or EGFR × TP53 synergy.

**TABLE 2:** Conditional association statistics for CUL3 mutation enrichment in the EGFR/TP53 genomic background. Two-layer Fisher’s exact testing and a multivariate logistic regression model evaluating CUL3 mutation as a function of EGFR-oncogenic and TP53 mutation status, including their interaction.

Test	Predictor	Odds ratio	95% CI	P-value	FDR adjusted P
**Fisher’s exact** (layer 1: Full NSCLC)	TP53 vs. CUL3 co-occurrence	2.81	2.36–3.36	2.04 × 10^−35^	4.08 × 10^−35^
**Fisher’s exact** (layer 2: EGFR-oncogenic subset)	TP53 vs. CUL3 co-occurrence	2.51	1.56–4.16	4.14 × 10^−5^	4.14 × 10^−5^
**Logistic regression** (CUL3 ∼ EGFR x TP53)	EGFR-oncogenic (main effect)	0.65	0.42–1.01	5.48 × 10^−2^	7.30 × 10^−2^
**Logistic regression** (CUL3 ∼ EGFR x TP53)	TP53 mutation (main effect)	2.92	2.42–3.53	4.68 × 10^−29^	9.35 × 10^−29^
**Logistic regression** (CUL3 ∼ EGFR x TP53)	EGFR x TP53 interaction	0.86	0.52–1.41	5.47 × 10^−1^	5.47 × 10^−1^

Logistic regression model: logit (P [CUL3-Mut]) ∼ EGFR_Onco + TP53_Mut + EGFR_Onco × TP53_Mut, fit using R glm () with family = binomial. The non-significant interaction term (P = 5.47 × 10^−1^) and significant TP53 main effect (P = 4.68 × 10^−29^) together demonstrate that CUL3 enrichment is driven by the TP53-altered microenvironment without an additional EGFR × TP53 synergistic effect. P-values are adjusted using the Benjamini-Hochberg false discovery rate procedure.

The magnitude of enrichment further indicates that this relationship is not subtle but reflects a meaningful biological signal. Such enrichment patterns are often indicative of selective pressure during tumor evolution. In this case, the data are consistent with a model in which TP53-deficient tumors create a permissive environment in which additional regulatory perturbations, such as those affecting CUL3, are tolerated or favoured; however, the Fisher analysis alone cannot distinguish between TP53-driven enrichment and EGFR × TP53 synergistic enrichment, motivating the interaction model presented in Section 3.3.

Because AACR GENIE contributes the largest number of samples to the pooled dataset and accounts for the majority of triple-positive cases, a cohort-bias sensitivity analysis was performed by removing the GENIE contribution and re-testing the association (R2 cohort-robustness analysis). At the population level (Layer 1), the CUL3–TP53 co-occurrence remained strong and highly significant after excluding GENIE (OR = 2.99, p = 5.1 × 10^−14^), confirming that the primary association is not an artefact of a single dominant cohort. Within the EGFR-oncogenic subset (Layer 2), by contrast, the leave-GENIE-out estimate attenuated substantially (OR ≈ 1.21, p ≈ 0.80), reflecting the small number of EGFR-oncogenic CUL3-mutant events contributed by the non-GENIE cohorts. This layer-specific pattern is itself informative: it indicates that the robust, cohort-independent signal resides in the general (TP53-driven) CUL3–TP53 association rather than in an EGFR-restricted niche, consistent with the non-significant EGFR × TP53 interaction reported below.

### 3.3 Non-additive interaction between EGFR and TP53

Logistic regression with an EGFR × TP53 interaction term confirmed that CUL3 enrichment is driven by the TP53 main effect rather than by EGFR × TP53 synergy. The TP53 main effect was strongly significant (OR = 2.92, 95% CI 2.42–3.53; p = 4.68 × 10^−29^; FDR = 9.35 × 10^−29^), indicating that TP53-mutant patients are nearly three-fold more likely to carry a concurrent CUL3 mutation than TP53-wild-type patients after adjusting for EGFR status. The EGFR main effect did not reach statistical significance (OR = 0.65, 95% CI 0.42–1.01; p = 0.055; FDR = 0.073), and critically, the EGFR × TP53 interaction term was non-significant (OR = 0.86, 95% CI 0.52–1.41; p = 0.547; FDR = 0.547), providing no evidence for EGFR-dependent modulation of the TP53–CUL3 association beyond an additive effect (Table 2; Figure 1E).

**FIGURE 1:**
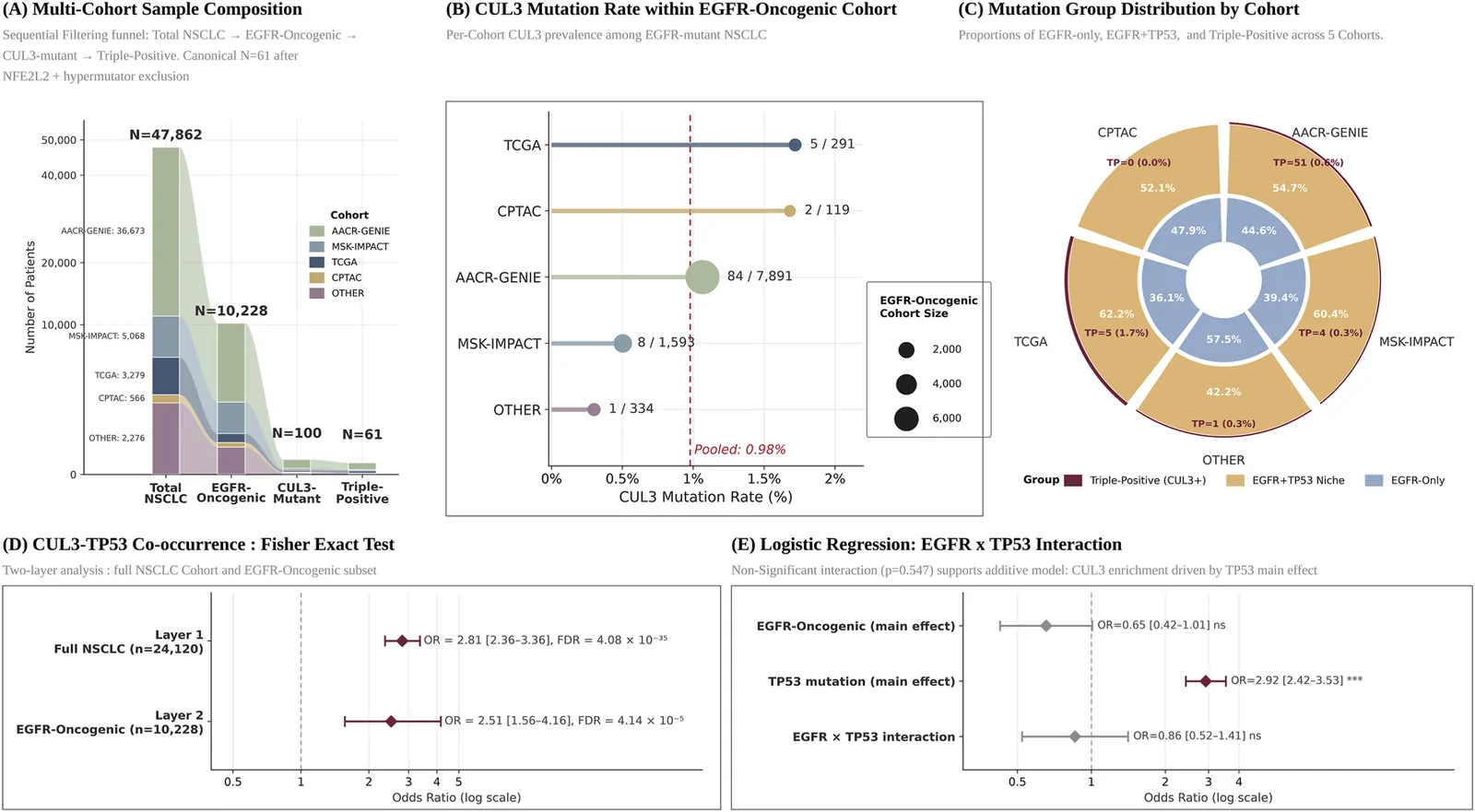
Multi-cohort genomic landscape and conditional enrichment of CUL3 mutations in EGFR-oncogenic NSCLC. **(A)** Sample composition funnel across five repositories (n = 47,862 total) through EGFR-oncogenic (n = 10,228), CUL3-mutant (n = 100), and canonical Triple-Positive (n = 61) after NFE2L2 and hypermutator exclusion. **(B)** Per-cohort CUL3 mutation rate within the EGFR-oncogenic background; pooled rate 0.98%. **(C)** Nested donut chart of the proportions of EGFR-Only (inner ring), EGFR + TP53 (outer ring) and Triple-Positive (maroon rim, with per-cohort counts; total n = 61) across the five cohorts. **(D)** Two-layer Fisher exact test: Layer 1 (all NSCLC, n = 24,120) OR = 2.81 (FDR = 4.08 × 10^−35^); Layer 2 (EGFR-oncogenic, n = 10,228) OR = 2.51 (FDR = 4.14 × 10^−5^). **(E)** Logistic regression confirming CUL3 enrichment is driven by TP53 main effect (OR = 2.92, p = 4.68 × 10^−29^); EGFR × TP53 interaction non-significant (OR = 0.86, p = 0.547).

The stability of these downstream analyses is constrained by a small event count, and this limitation warrants explicit discussion. Although the full NSCLC cohort is large, only 100 CUL3-mutant cases occur within the 10,228 EGFR-oncogenic samples, and the canonical triple-positive cohort comprises 61 patients after confounder exclusion; subgroup analyses (TMB, metastatic origin, histology) therefore rest on modest denominators and should be regarded as exploratory. To guard against small-sample instability in the interaction model, the logistic analysis was repeated using Firth’s penalised-likelihood correction, which is robust to sparse-data and separation bias. The Firth-corrected EGFR × TP53 interaction remained non-significant (OR = 0.85, p = 0.52), confirming that the null interaction is not an artefact of sparse triple-positive counts, and reinforcing the conclusion that the association is TP53-driven rather than EGFR-dependent.

The non-significant interaction term carries an important interpretive consequence: although CUL3 mutations co-occur preferentially with TP53 alterations, this preference is not amplified by the additional presence of an oncogenic EGFR mutation. The combined evidence is consistent with TP53 loss creating the dominant selective context for CUL3-mutant clonal expansion, with EGFR oncogenic signalling acting as the disease-defining background within which this TP53-dependent association is observed, rather than as a co-modifier of the association strength.

This pattern reframes the role of CUL3 in EGFR-driven NSCLC: rather than emerging from an EGFR × TP53 synergistic niche, CUL3 mutations appear to be selected within the broader population of TP53-altered tumors, of which EGFR-oncogenic NSCLC is one biologically defined subset. Such patterns are characteristic of context-dependent modifiers whose selection is shaped by a single dominant genomic event (here, TP53 loss) rather than by combinatorial driver synergy. The interaction model therefore reinforces an interpretation of CUL3 as a conditional modifier within the EGFR/TP53-altered genomic framework rather than an obligate triple-driver event.

To address whether the TP53-dependent enrichment of CUL3 mutations is specific to the EGFR-oncogenic background or generalises across NSCLC driver contexts, the association was re-examined within tumors stratified by their principal oncogenic driver. In a curated, non-overlapping cohort (n = 9,398) in which EGFR, KRAS, ROS1, and MET driver status could be jointly resolved with TP53 status, the CUL3–TP53 association was strongest and most statistically robust in the driver-negative subgroup (OR = 6.23, p = 1.8 × 10^−12^), while remaining directionally positive but not individually significant within the EGFR (OR = 0.86, p = 0.79), KRAS (OR = 1.53, p = 0.14), ROS1 (OR = 4.61, p = 0.19), and MET (OR = 4.83, p = 0.18) subgroups; the pooled association across all contexts was OR = 2.75 (p = 7.2 × 10^−10^). The absence of an EGFR-specific signal, together with the dominant driver-negative enrichment, indicates that the CUL3–TP53 association is a general feature of TP53-altered NSCLC rather than a property of the EGFR background specifically.

This interpretation was corroborated in the independently released AACR GENIE v20.0 cohort (n = 24,646 with confirmed CUL3 coverage), analysed here as revision-stage large-cohort validation of the driver-context question. In this larger dataset the enrichment was again maximal in the driver-negative subgroup (OR = 3.75, p = 6.2 × 10^−19^) and, at the increased sample size, additionally reached significance within the EGFR (OR = 2.22, p = 1.7 × 10^−3^) and MET (OR = 3.62, p = 4.1 × 10^−3^) subgroups, with a borderline KRAS signal (OR = 1.45, p = 0.045) that did not survive multiple-testing correction. Across both datasets the enrichment is consistently strongest in driver-negative, TP53-defined tumors, reinforcing the interpretation that TP53 alteration rather than any specific co-occurring driver provides the dominant selective context for CUL3 mutation.

### 3.4 NRF2-axis confound assessment

Within the pre-sensitivity triple-positive group (N = 68 after hypermutator exclusion), co-mutation rates of canonical NRF2-axis genes were 22.1% for KEAP1 (15/68), 20.6% for STK11/LKB1 (14/68), and 10.3% for NFE2L2 (7/68). Applying the pre-specified 15% confound threshold, KEAP1 and STK11 exceeded the cutoff and were designated for sensitivity analysis, whereas NFE2L2 fell below the threshold but was nonetheless excluded from the canonical cohort because activating NFE2L2 variants directly mimic the CUL3-loss phenotype at the transcriptional level (Figure 2A). Relative to the EGFR + TP53 niche, KEAP1 and STK11 were also significantly enriched in the canonical triple-positive cohort (KEAP1 21.3% vs. 3.1%, OR = 8.59, FDR = 9.9 × 10^−8^; STK11 14.8% vs. 3.6%, OR = 4.66, FDR = 3.6 × 10^−4^; Figure 3D).

**FIGURE 2:**
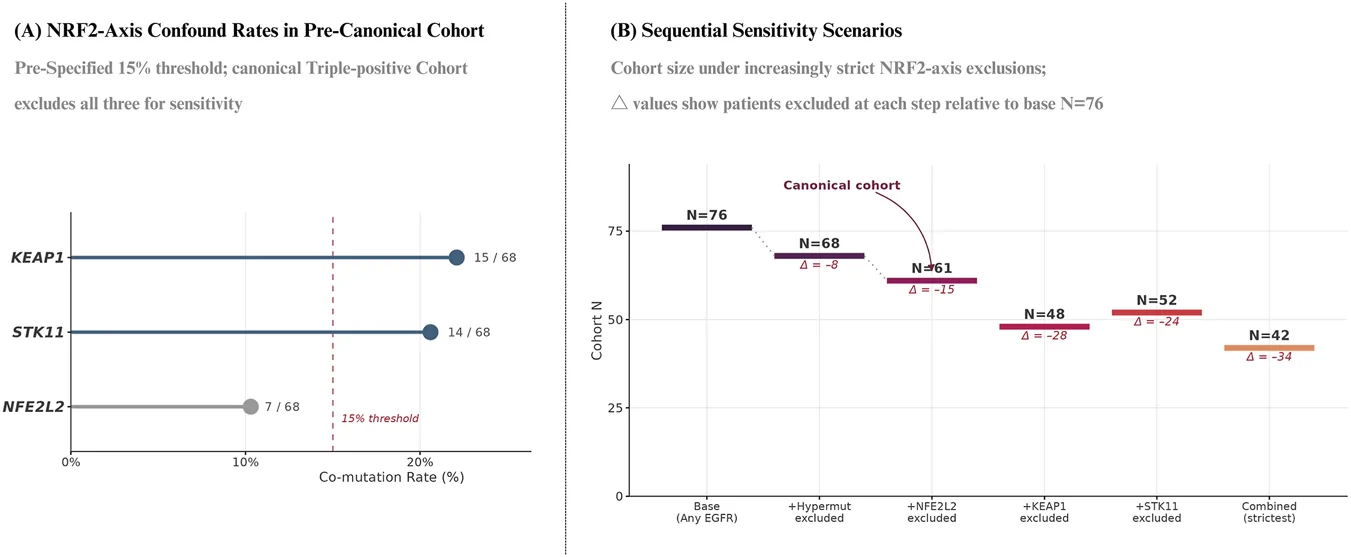
NRF2-axis confound assessment and sensitivity analysis of the canonical Triple-Positive cohort. **(A)** Co-mutation rates of KEAP1 (22.1%), STK11 (20.6%), and NFE2L2 (10.3%) in the pre-sensitivity cohort (n = 68); red dashed line = pre-specified 15% threshold. **(B)** Sequential cohort attrition across six NRF2-axis exclusion scenarios (N = 76 → 61 canonical → 42 strictest); Δ values indicate cumulative exclusions from base N = 76.

**FIGURE 3:**
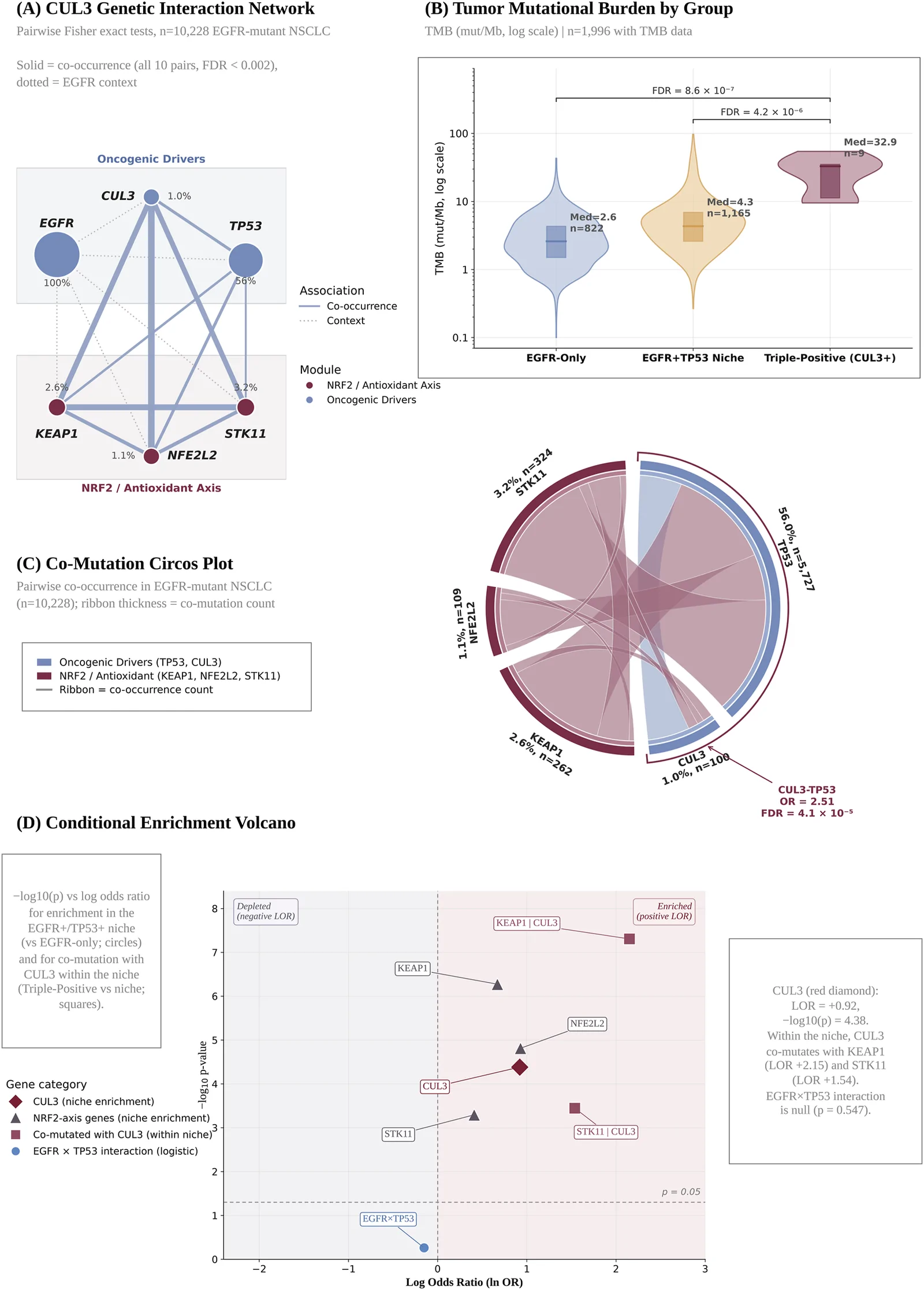
Genomic severity and co-mutational architecture of the EGFR/TP53/CUL3 Triple-Positive subgroup. **(A)** Pairwise co-mutation network in the EGFR-oncogenic cohort (n = 10,228); node size ∝ mutation frequency, nodes grouped into oncogenic-driver and NRF2/antioxidant modules, and line width ∝ log odds ratio; all ten gene pairs co-occur (Fisher exact, FDR <0.002), with EGFR context links dotted. **(B)** Tumour mutational burden by group (n = 1,996); medians: Triple-Positive 32.9 (n = 9), EGFR + TP53 4.3 (n = 1,165), EGFR-Only 2.6 (n = 822) mut/Mb; Triple-Positive versus EGFR + TP53 FDR = 4.2 × 10^−6^ and versus EGFR-Only FDR = 8.6 × 10^−7^ (two-sided Mann–Whitney U, Benjamini–Hochberg). **(C)** Chord diagram of pairwise co-mutation counts; ribbon width ∝ number of co-mutated samples; CUL3–TP53 highlighted (n = 76; OR = 2.51, FDR <1 × 10^−4^). **(D)** Conditional enrichment volcano (−log_10_ p versus ln OR). Circles and the CUL3 diamond show enrichment of each gene in the EGFR + TP53 Niche relative to EGFR-Only tumours (CUL3 ln OR = +0.92, p = 4.1 × 10^−5^); squares show co-mutation with CUL3 within the Niche (canonical Triple-Positive, n = 61, versus EGFR + TP53, n = 5,666): KEAP1 OR = 8.59 (95% CI 4.19–16.48; FDR = 9.9 × 10^−8^) and STK11 OR = 4.66 (95% CI 1.99–9.71; FDR = 3.6 × 10^−4^); the EGFR × TP53 interaction term from the logistic model is shown for reference (p = 0.547). NFE2L2 was not tested within the Niche because NFE2L2-mutant cases were excluded from the canonical cohort.

Sequential exclusion of NRF2-axis confounders (NFE2L2, KEAP1, and STK11) reduced the triple-positive cohort from 76 (base cohort) through 68 (after hypermutator exclusion), 61 (canonical, after NFE2L2 exclusion), 48 (after additional KEAP1 exclusion), and 52 (after additional STK11 exclusion), to 42 in the strictest combined NRF2-axis-clean scenario (Figure 2B; Supplementary Table S1). Across all six exclusion scenarios, the underlying pattern of CUL3 enrichment within the EGFR + TP53 background was preserved, indicating that the observed niche is not solely driven by canonical NRF2-axis lesions.

The persistence of the CUL3 enrichment signal after sequential confounder exclusion demonstrates that the observation is not a downstream reflection of canonical NRF2 pathway alterations, which are themselves potent drivers of oxidative-stress adaptation. By systematically removing these confounders, the analysis isolates the residual CUL3-specific contribution and supports an interpretation of CUL3 perturbation as an independent, non-canonical route to NRF2-axis dysregulation. This distinction is mechanistically important and frames the downstream transcriptomic and structural analyses, which seek to characterise the functional consequences of CUL3-specific perturbation in tumors free of overt NRF2-axis lesions.

### 3.5 Elevated tumor mutational burden in triple-positive tumors

Tumor mutational burden (TMB) analysis revealed a marked elevation in the triple-positive group relative to both EGFR-only and EGFR + TP53 cohorts. Among the triple-positive patients with available TMB data (n = 9 of 61, 15% data completeness), median TMB was 32.9 mutations/Mb, approximately 7.7-fold higher than the EGFR + TP53 Niche (median 4.3 mutations/Mb) and 12.7-fold higher than EGFR-only tumors (median 2.6 mutations/Mb) (Mann-Whitney U, FDR-adjusted p = 4.2 × 10^−6^ versus the EGFR + TP53 Niche and 8.6 × 10^−7^ versus EGFR-only; Figure 3B; Supplementary Figure S2B). This result must be interpreted with substantial caution. TMB was available for only 9 of 61 triple-positive patients (15%), and TMB availability is not random across the constituent cohorts panel-based assays (e.g., MSK-IMPACT) report TMB routinely whereas other datasets do not, so the triple-positive TMB sample is subject to ascertainment bias toward panel-sequenced tumors. With n = 9, the comparison is highly sensitive to the underlying distribution and to the choice of test, and the FDR-adjusted p-value should be regarded as descriptive rather than as robust formal inference. The direction of the effect is nonetheless concordant with the well-established association between TP53 loss and elevated mutational burden, and is reported here as a hypothesis-generating observation requiring confirmation in cohorts with complete, uniformly ascertained TMB data.

The elevated TMB in triple-positive tumors is consistent with the known consequences of TP53 loss on DNA damage response and genomic stability. The further increase observed beyond the EGFR + TP53 Niche group is hypothesis-generating: it may reflect additional disruption of cellular stress-regulation circuits or accelerated mutation accumulation in tumors that have evolved CUL3-mediated NRF2 axis bypass. High mutational burden is clinically relevant as a determinant of immunotherapy eligibility (TMB-High threshold ≥10 mutations/Mb), and several of the triple-positive samples in our analysis exceeded this threshold, raising the possibility that this subgroup may exhibit differential response to immune checkpoint blockade.

### 3.6 Metastatic enrichment as a contextual severity indicator

Biopsy origin annotation was available for 8,073 patients across the three groups. Metastatic-site biopsies comprised 45.2% of triple-positive samples, 51.4% of EGFR + TP53 Niche samples, and 35.6% of EGFR-only samples. The chi-squared test did not reach statistical significance for the triple-positive comparisons (Triple vs. EGFR-only p = 0.25; Triple vs. Niche p = 0.52), reflecting the limited triple-positive denominator with known biopsy site. The proportion of metastatic biopsies in the triple-positive group is intermediate between the EGFR-only and EGFR + TP53 groups, with the EGFR + TP53 niche itself displaying the highest metastatic enrichment, indicating that biopsy origin alone does not stratify triple-positive tumors as more advanced than the broader EGFR + TP53 niche.

Together, the genomic analyses define CUL3 mutations as a rare but reproducible feature of EGFR-driven NSCLC, conditionally enriched in the TP53-altered background, robust to NRF2-axis confounders, and accompanied by elevated mutational burden. To assess whether this genomic niche carries functional consequences at the transcriptional level, we performed a transcriptomic analysis of NRF2 pathway activation, immune microenvironment composition, drug-metabolism gene expression, and neddylation-pathway co-expression in CUL3-mutant tumors.

Because the pooled cohort combines lung adenocarcinoma (LUAD) and lung squamous cell carcinoma (LUSC), the CUL3–TP53 association was additionally examined after stratification by histological subtype to determine whether the pooled signal is attributable to a single dominant histology. Within LUAD, the association remained significant (OR = 2.18, p = 6.3 × 10^−3^), and its robustness was confirmed in the larger AACR GENIE v20.0 LUAD subset (n = 18,159; OR = 2.10, p = 1.0 × 10^−10^). LUSC contributed substantially fewer CUL3-mutant events, consistent with the predominance of LUAD among EGFR-oncogenic and TP53-altered NSCLC; the directionally concordant but underpowered LUSC estimate precludes a definitive subtype-specific conclusion. The persistence of the association within LUAD indicates that the pooled result is not an artefact of histological mixing and identifies adenocarcinoma as the principal histological context in which the CUL3–TP53 relationship is observed. On the basis of the observed transcriptomic NRF2 activation and the LUAD-restricted genomic enrichment, CUL3-mutant LUAD tumors are predicted to display elevated nuclear NRF2 immunostaining and increased NRF2 target-protein expression (e.g., NQO1, GCLC) relative to CUL3–wild-type LUAD.

### 3.7 NRF2 pathway activation in CUL3-mutant tumors

Across three independent canonical NRF2 pathway gene signatures, CUL3-mutant tumors displayed significantly elevated activation scores compared with CUL3 wild-type samples (n = 31 vs. 89; Figure 4 and Table 3). The MSigDB SINGH_NFE2L2_TARGETS signature (14 genes; Singh et al., 2008) showed strong separation in the full cohort (mean ΔZ = +0.48; Wilcoxon p = 1.1 × 10^−3^, FDR = 3.3 × 10^−3^) with consistent direction in the held-out 30% validation split (p = 1.1 × 10^−3^, FDR = 1.9 × 10^−3^; Supplementary Figure S2A). The Best NSCLC NRF2 signature (19 genes; Best et al., 2018) confirmed the result (mean ΔZ = +0.51; full cohort p = 2.4 × 10^−3^, FDR = 3.6 × 10^−3^; validation p = 1.3 × 10^−3^, FDR = 1.9 × 10^−3^). The HALLMARK_REACTIVE_OXYGEN_SPECIES_PATHWAY (49 genes) was also significant, with a smaller effect (mean ΔZ = +0.24; full cohort p = 1.3 × 10^−2^, FDR = 1.3 × 10^−2^; validation p = 2.0 × 10^−3^, FDR = 2.0 × 10^−3^) (Liberzon et al., 2015). The concordance of effect direction and magnitude across three orthogonally curated gene panels argues against signature-specific artefact and indicates a transcriptional program consistent with NRF2-driven oxidative-stress adaptation.

**FIGURE 4:**
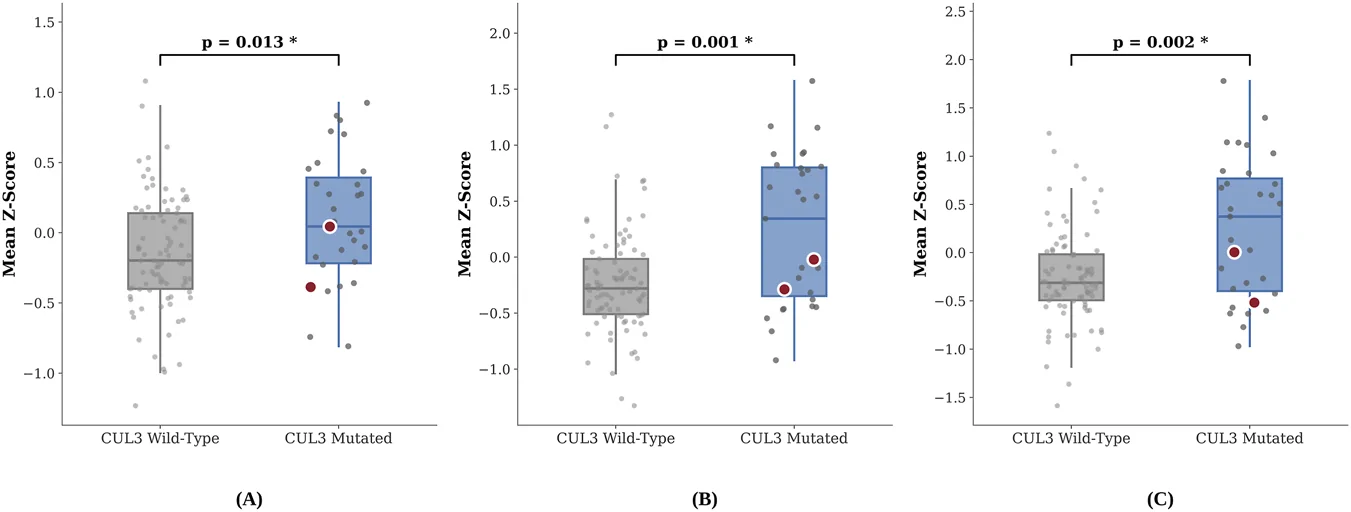
CUL3 loss activates the NRF2 pathway across three independent canonical gene signatures. Mean Z-score activation in CUL3 wild-type (grey, n = 89) versus CUL3-mutant (blue, n = 31, including 2 Triple-Positive patients in dark red) TCGA LUAD/LUSC. **(A)** HALLMARK_ROS (49 genes; p = 0.013, FDR = 0.013). **(B)** SINGH_NFE2L2_TARGETS (14 genes; p = 1.1 × 10^−3^, FDR = 3.3 × 10^−3^). **(C)** BEST_NRF2_TARGETS (19 genes; p = 2.4 × 10^−3^, FDR = 3.6 × 10^−3^). Two-sided Wilcoxon rank-sum test; Benjamini–Hochberg FDR across the three signatures.

**TABLE 3:** Transcriptomic signatures of NRF2 pathway activation, immune microenvironment, drug metabolism, and neddylation co-expression in CUL3-mutant tumors. Per-signature and per-gene statistics from the EGFR-eligible TCGA cohort (n = 120; 89 wild-type, 31 CUL3-mutant including 2 triple-positive), with Wilcoxon rank-sum tests for group comparisons and Spearman rank correlations against the canonical SINGH_NFE2L2_TARGETS score. All gene-set definitions traced to canonical published sources.

Analysis module	Gene or signature	Effect size	Group/Cohort	P-value	FDR adjusted P
NRF2 pathway activation	HALLMARK_ROS_PATHWAY (49 genes)	ΔZ = +0.24	CUL3-mut > WT	1.26 × 10^−2^	1.26 × 10^−2^
	SINGH_NFE2L2_TARGETS (14 genes; M2662)	ΔZ = +0.48	CUL3-mut > WT	1.11 × 10^−3^	3.32 × 10^−3^
	BEST_NRF2_TARGETS (19 genes)	ΔZ = +0.51	CUL3-mut > WT	2.42 × 10^−3^	3.62 × 10^−3^
Immune microenvironment	Proliferation	Δ = +0.141	0.230 vs. 0.089	4.90 × 10^−7^	4.41 × 10^−6^
	Interferon	Δ = −0.044	0.269 vs. 0.312	1.82 × 10^−2^	8.17 × 10^−2^ (ns)
	Neutrophils	Δ = −0.018	0.199 vs. 0.216	4.72 × 10^−2^	1.42 × 10^−1^ (ns)
	Translation	Δ = +0.005	0.520 vs. 0.515	1.23 × 10^−1^	2.38 × 10^−1^ (ns)
	Monocytes	Δ = −0.018	0.253 vs. 0.271	1.59 × 10^−1^	2.38 × 10^−1^ (ns)
	T cells	Δ = −0.029	0.016 vs. 0.045	1.79 × 10^−1^	2.38 × 10^−1^ (ns)
	Macrophages	Δ = −0.027	0.223 vs. 0.251	1.85 × 10^−1^	2.38 × 10^−1^ (ns)
	NK cells	Δ = +0.013	−0.286 vs. −0.299	3.00 × 10^−1^	3.37 × 10^−1^ (ns)
	B cells	Δ = −0.022	−0.138 vs. −0.116	4.57 × 10^−1^	4.57 × 10^−1^ (ns)
	Plasma cells	—	Insufficient gene coverage	—	—
Drug metabolism gene	*UGT1A1*	ΔZ = +1.32	−0.34 vs. −1.65	1.09 × 10^−5^	1.97 × 10^−4^
	*UGT1A9*	ΔZ = +2.17	+0.46 vs. −1.71	2.02 × 10^−3^	1.21 × 10^−2^
	*CYP3A5*	ΔZ = −0.89	−0.78 vs. +0.11	9.98 × 10^−5^	8.98 × 10^−4^
	*UGT2B7*	ΔZ = −0.53	−0.94 vs. −0.41	9.19 × 10^−3^	4.14 × 10^−2^
Neddylation pathway Co-expression	*NAE1*	ρ = +0.264	vs. singh NRF2 score	3.52 × 10^−3^	7.20 × 10^−3^
	*UBA3*	ρ = +0.261	vs. singh NRF2 score	4.00 × 10^−3^	7.20 × 10^−3^
	*UBE2F*	ρ = +0.246	vs. singh NRF2 score	6.71 × 10^−3^	1.01 × 10^−2^
	*RBX1*	ρ = +0.280	vs. singh NRF2 score	1.93 × 10^−3^	5.80 × 10^−3^
	*KEAP1*	ρ = +0.309	vs. singh NRF2 score	6.04 × 10^−4^	2.72 × 10^−3^
	*NFE2L2*	ρ = +0.388	vs. singh NRF2 score	1.22 × 10^−5^	1.10 × 10^−4^

Loss or perturbation of CUL3 function would be predicted to stabilise NRF2 and elevate transcription of downstream antioxidant and cytoprotective genes; the observed transcriptomic pattern matches this prediction and provides indirect evidence of altered CRL3 complex activity in CUL3-mutant tumors at the population level.

### 3.8 Proliferative and immune microenvironment changes

Single-sample gene set enrichment analysis (ssGSEA) of the canonical imSig immune-cell signatures identified a markedly elevated proliferation signature in CUL3-mutant tumors (mean ssGSEA score 0.230 versus 0.089 in wild-type; Wilcoxon FDR = 4.4 × 10^−6^; Figure 5A and Table 3) (Nirmal et al., 2018). Proliferation was the only signature reaching FDR significance and exhibited the largest effect size in the Phase B analysis, consistent with NRF2-driven upregulation of cell-cycle and biosynthetic programs downstream of antioxidant pathway activation.

**FIGURE 5:**
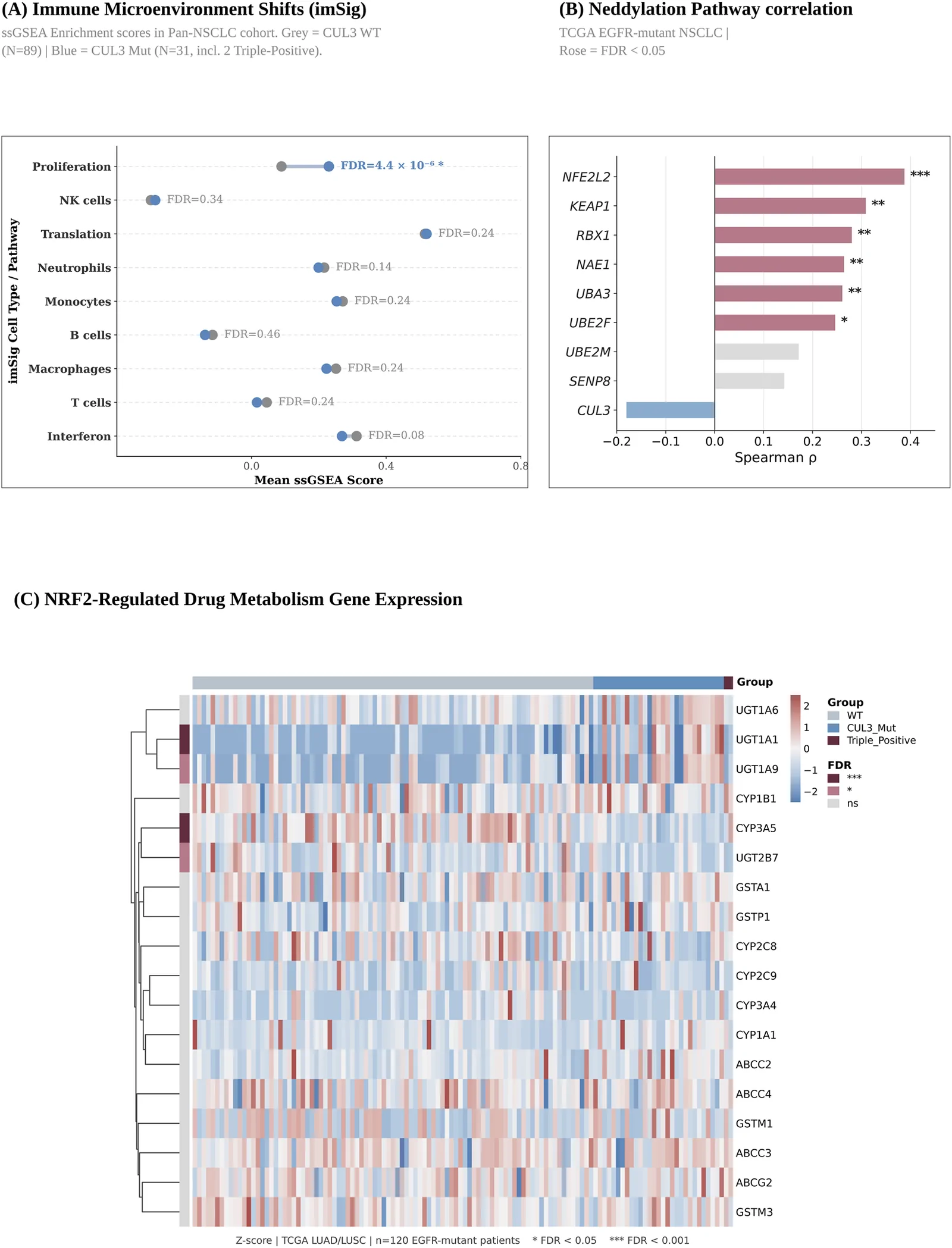
Immune microenvironment, neddylation co-expression, and drug metabolism reprogramming in CUL3-mutant NSCLC. **(A)** ssGSEA imSig scores (n = 120; 89 wild-type, 31 CUL3-mutant), shown as group means; Proliferation is the only FDR-significant category (FDR = 4.4 × 10^−6^); Interferon shows a near-significant reduction (FDR = 0.082). **(B)** Spearman correlations of neddylation pathway genes with SINGH_NFE2L2_TARGETS score; NFE2L2 (ρ = 0.388), KEAP1 (ρ = 0.309), RBX1, NAE1, UBA3, UBE2F reach FDR significance. **(C)** Patient-level heatmap of 18 NRF2-regulated drug metabolism genes (n = 120; columns grouped as wild-type, CUL3-mutant and Triple-Positive); UGT1A1 (FDR = 1.97 × 10^−4^) and CYP3A5 (FDR = 8.98 × 10^−4^) show strongest group differences.

The Interferon-response signature, which captures type-I interferon-stimulated gene expression, showed a directional reduction in CUL3-mutant tumors approaching significance (FDR = 0.082; nominal p = 0.018; Figure 5A). This finding is consistent with the established suppressive effect of NRF2 activation on type-I interferon signalling and with the immune-cold phenotype of KEAP1- and STK11-altered lung adenocarcinomas (Skoulidis et al., 2018; Olagnier et al., 2020).

Among the remaining canonical immune-cell signatures, all showed directional differences consistent with reduced immune infiltration in CUL3-mutant tumors, but none reached FDR significance after multiple-testing correction. Macrophages, monocytes, neutrophils, T cells, and B cells trended lower in CUL3-mutant tumors, while NK cells trended slightly higher (Table 3; Supplementary Table S2). Plasma cells were excluded from the analysis owing to insufficient gene-symbol coverage in the TCGA RSEM matrix (4 of 14 imSig plasma-cell genes recovered). The combination of significantly elevated proliferation, dampened interferon response, and directionally reduced immune infiltration is consistent with a tumor state combining intrinsic growth advantage with diminished immune surveillance, motivating future investigation in immunotherapy-treated cohorts.

### 3.9 Altered drug metabolism gene expression in CUL3-mutant tumors

Among the 18 NRF2-regulated drug metabolism genes profiled, four reached FDR significance in the CUL3-mutant versus wild-type comparison (Figure 5C; Table 3; Supplementary Table S3). The two largest changes were observed in the Phase II glucuronidation enzymes UGT1A1 (ΔZ = +1.32; p = 1.09 × 10^−5^, FDR = 1.97 × 10^−4^) and UGT1A9 (ΔZ = +2.17; p = 2.02 × 10^−3^, FDR = 1.21 × 10^−2^), both of which are canonical NRF2 transcriptional targets via ARE-mediated regulation. Conversely, the Phase I oxidative enzyme CYP3A5 (ΔZ = −0.89; p = 9.98 × 10^−5^, FDR = 8.98 × 10^−4^) and the Phase II enzyme UGT2B7 (ΔZ = −0.53; p = 9.19 × 10^−3^, FDR = 4.14 × 10^−2^) were significantly downregulated in CUL3-mutant tumors. The combination of selective UGT induction (UGT1A1, UGT1A9) and CYP3A5 suppression suggests a coordinated metabolic reprogramming away from oxidative Phase I metabolism toward conjugative Phase II clearance, which is biologically consistent with NRF2-driven UGT activation. We note that several of these enzymes (UGT1A1, UGT1A9, CYP3A5) participate more broadly in xenobiotic metabolism; however, these data are transcriptional associations only and do not measure enzyme activity, protein abundance, or drug disposition.

### 3.10 Neddylation pathway coupling with NRF2 activity

To establish a transcriptomic bridge between NRF2 activation and the neddylation-dependent functional state of CRL3 (the mechanistically relevant context for downstream structural modelling), expression of nine neddylation pathway components was profiled and tested for both group-level differences and correlation with the SINGH_NFE2L2_TARGETS activation score (Figure 5B; Table 3). At the group level, UBA3, the catalytic E1 subunit of the NEDD8-activating enzyme, showed significant upregulation in CUL3-mutant tumors (Wilcoxon FDR = 4.35 × 10^−2^), suggesting compensatory upregulation of NEDD8 conjugation machinery in tumors with reduced CRL3 neddylation efficiency. More strikingly, Spearman rank correlation across all 120 patients revealed coherent positive associations between the NRF2 activation score and multiple core neddylation components: NAE1 (ρ = 0.264, FDR = 7.20 × 10^−3^), UBA3 (ρ = 0.261, FDR = 7.20 × 10^−3^), UBE2F (ρ = 0.246, FDR = 1.01 × 10^−2^), RBX1 (ρ = 0.280, FDR = 5.80 × 10^−3^), KEAP1 (ρ = 0.309, FDR = 2.72 × 10^−3^), and NFE2L2 itself (ρ = 0.388, FDR = 1.10 × 10^−4^).

The NFE2L2 correlation is expected because NFE2L2 mRNA is part of the NRF2 transcriptional output; however, the coherent positive correlation of NEDD8 E1 (NAE1, UBA3), the alternative E2 (UBE2F), and the CRL RING substrate (RBX1) with NRF2 activation provides transcriptomic evidence that tumors with elevated NRF2 output engage the neddylation machinery at higher levels, consistent with the active CRL3 conformation being the dominant biological context. This finding directly motivates the structural-mechanistic strategy of prioritising the neddylation-active CUL3:KEAP1:NEDD8 system for molecular dynamics analysis, since it is in this conformation that CUL3 missense variants are most likely to exert dominant-negative perturbation of NRF2 turnover.

### 3.11 Structural impact of CUL3 mutation on the CUL3–KEAP1 scaffold

Structural modeling of CUL3:KEAP1 complex made it possible to measure the perturbation of mutation in the Cullin scaffold among the inactive and neddylated (active) states. L321F was selected as the primary structural exemplar on the basis of its localisation within a structurally interpretable, buried helical region of the N-terminal cullin-repeat domain and its relevance to the KEAP1/NEDD8 system. SIFT and polyphen2 predicted a score of 0.06 and 1.00 respectively, which are borderline tolerance to evolutionary conservation but a strong probability of structural impact. Mapping of the mutation onto the CUL3 scaffold (Figures 6A,C) revealed its localization within a structured, buried region of the N-terminal cullin-repeat domain, where substitution of leucine with the bulkier phenylalanine introduces local packing alterations. This substitution is expected to influence the conformational stability of the scaffold and potentially modulate the positioning of adaptor-interacting regions. In the context of the neddylated state, such perturbations may alter the conformational dynamics required for efficient CRL3 activation.

**FIGURE 6:**
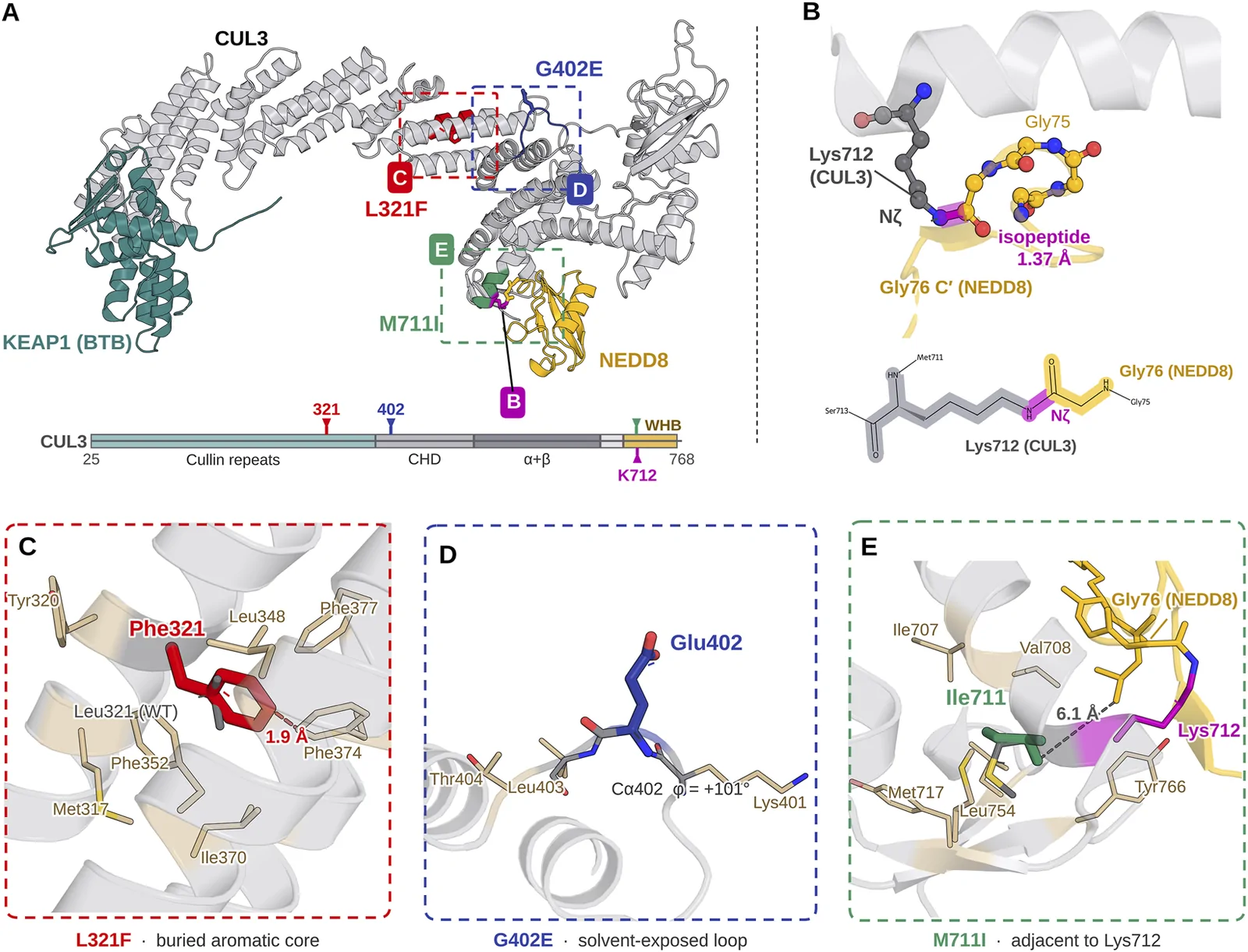
Structural context of the three modelled CUL3 missense variants in the neddylated CUL3:KEAP1 complex. **(A)** Energy-minimised model of CUL3 (grey), the KEAP1 BTB domain (teal) and NEDD8 (gold), with L321F (red), G402E (blue) and M711I (green) highlighted; dashed boxes mark the regions enlarged in panels C–E. The track below shows the CUL3 domain architecture, with each variant in a different domain and the Lys712 neddylation site marked. **(B)** Isopeptide bond (1.37 Å) between the CUL3 Lys712 Nζ and the C-terminal Gly76 of NEDD8, with a two-dimensional scheme below. **(C–E)** Wild-type (grey) and variant (coloured) side chains superposed, with neighbouring residues in wheat. **(C)** L321F lies in a buried aromatic core, with the phenyl ring 1.9 Å from Phe374. **(D)** G402E introduces a negative charge into a solvent-exposed glycine loop (φ = +101°). **(E)** M711I is near-isosteric and buried next to Lys712, without direct NEDD8 contact (≥6.1 Å). Phe321 and Ile711 were modelled as lowest-strain rotamers; the corresponding neddylated-state dynamics are shown in Figure 8.

These annotations provide a structural model in which CUL3 mutations are not direct interface disruptive events, but rather supported by allosteric modulation of the scaffold, which is consistent with role of CUL3 as context-dependent modifier but not as primary driver. The structural framework recognized here forms a base for subsequent molecular dynamics analysis.

To place L321F within the broader mutational landscape of CUL3 and to address whether the structural inference is variant-specific, the spectrum of CUL3 missense variants observed across the cohorts was catalogued with respect to domain localisation on the canonical isoform (ENST00000264414; 768 aa) and scored with complementary *in silico* predictors. Recurrent and high-impact variants distributed across both the N-terminal cullin-repeat/adaptor-binding region and the C-terminal domain that harbours the K712 neddylation site, including G402E (n = 6; ESM-2 LLR −6.10, SIFT 0.03, PolyPhen-2 0.968), E375Q (n = 5; SIFT 0.00, PolyPhen-2 1.000), E424K (n = 5; SIFT 0.00, PolyPhen-2 0.982), M599R (n = 4; strongest predicted impact, ESM-2 LLR −10.05, PolyPhen-2 1.000), and M711I (n = 4), the last of which lies immediately adjacent to the K712 neddylation lysine. To provide an orthogonal, alignment-based assessment of variant impact, all catalogued variants were additionally scored with the ESM-2 protein language model; ESM-2 log-likelihood ratios correlated with both SIFT (Spearman ρ = +0.62, p = 0.013) and PolyPhen-2 (ρ = −0.62, p = 0.014), indicating convergent prioritisation across three methodologically independent predictors. This distribution indicates that CUL3 perturbation in NSCLC is not confined to a single domain and that multiple variants are predicted to be functionally consequential, motivating variant-resolved structural analysis beyond the single exemplar modelled in detail here; the local environments of G402E and M711I are shown in Figures 6D,E.

It should be emphasised that the molecular dynamics results describe the specific structural behaviour of the L321F substitution and are not, by themselves, evidence that all CUL3 missense variants act through the same allosteric mechanism. Variants situated in different domains, for example, those adjacent to the K712 neddylation site (M711I) versus those in the N-terminal adaptor-binding region may perturb CRL3 function through distinct structural routes.

### 3.12 Construction of the neddylated CUL3:KEAP1 Complex and Trajectory Analysis

To investigate the conformational dynamics of CUL3 perturbation in its mechanistically relevant active state, the neddylation-active CUL3:KEAP1 complex was constructed by introducing a covalent isopeptide bond between CUL3 K712 NZ and NEDD8 G76 C. Successful construction of the isopeptide bond was confirmed by stable convergence of energy minimisation in both the wild-type (Fmax = 866 kJ mol^-1^ nm^-1^ at 1,570 steps) and L321F mutant systems (Fmax = 899 kJ mol^-1^ nm^-1^ at 2,060 steps). Both systems remained thermodynamically stable through the equilibration protocol (NVT at 310 K; NPT at 310 K and 1 bar; minimal energy drift), and the K712 NZ–G76 C bond length was maintained at 1.34 ± 0.01 Å throughout equilibration, confirming the parametric correctness of the imposed covalent linkage. Production molecular dynamics simulations of 200 ns each were subsequently performed on the wild-type and L321F systems in both inactive and neddylation-active conformations.

Backbone RMSD analysis confirmed structural stability across all eight simulated systems throughout the production trajectories (200 ns; 196 ns for M711I + NEDD8) (Figures 7A, 8A). In the inactive state, both wild-type and L321F CUL3:KEAP1 complexes exhibited comparable mean RMSD values (WT: 0.577 ± 0.118 nm; L321F: 0.581 ± 0.113 nm), indicating that the substitution does not perturb CUL3 backbone integrity in the non-activated conformation. In the neddylation-active state, however, the L321F + NEDD8 complex displayed a lower mean CUL3 RMSD (0.489 ± 0.091 nm) relative to WT + NEDD8 (0.597 ± 0.155 nm), with a correspondingly more pronounced reduction observed for the KEAP1 chain (WT + NEDD8: 0.528 ± 0.119 nm; L321F + NEDD8: 0.309 ± 0.079 nm; chain-resolved data). The consistent reduction across both components of the neddylated complex argues against interface destabilisation and instead indicates that the L321F substitution constrains the complex in a more rigid conformation upon neddylation. In the inactive state, G402E and M711I CUL3:KEAP1 complexes remained stable (mean backbone RMSD 0.607 ± 0.157 nm and 0.492 ± 0.109 nm, respectively; Figure 7A). In the neddylated state, M711I + NEDD8 behaved comparably to WT + NEDD8 (0.585 ± 0.143 nm), whereas G402E + NEDD8 showed the highest backbone RMSD of any system (0.740 ± 0.174 nm) driven by a progressive drift over the trajectory rather than a single excursion (20 ns block means rising monotonically from 0.58 to 1.03 nm; Figure 8A).

**FIGURE 7:**
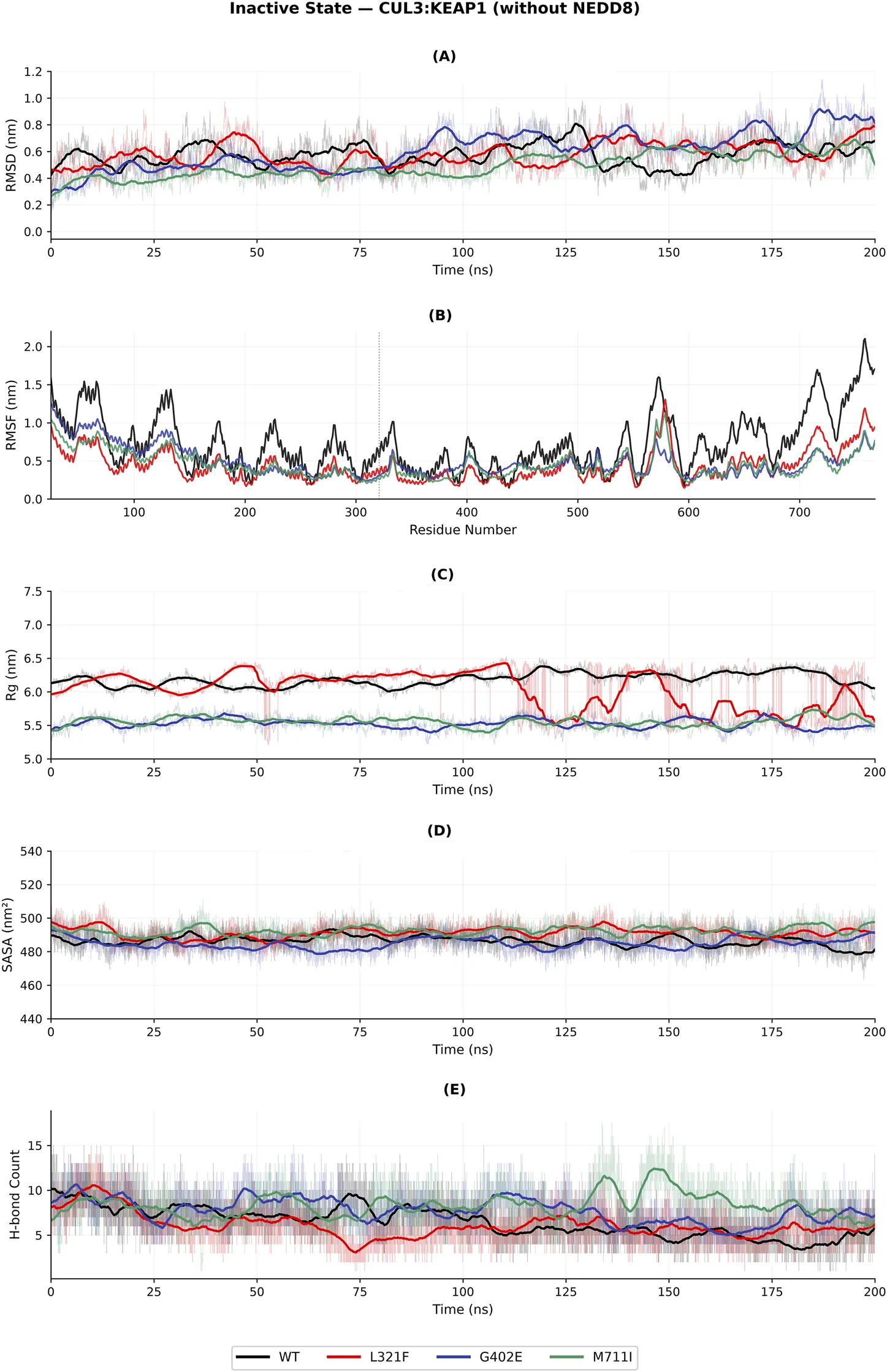
Molecular dynamics trajectory analysis inactive state (CUL3:KEAP1, without NEDD8) for four CUL3 variants. **(A)** CUL3 backbone RMSD over the 200 ns production trajectory; wild-type (black) 0.577 ± 0.118 nm; L321F (red) 0.581 ± 0.113 nm; G402E (blue) 0.607 ± 0.157 nm; M711I (green) 0.492 ± 0.109 nm. **(B)** Per-residue CUL3 Cα RMSF (wild-type 0.727 ± 0.375 nm; L321F 0.436 ± 0.204 nm; G402E 0.487 ± 0.192 nm; M711I 0.454 ± 0.172 nm); dotted vertical line indicates residue 321. **(C)** Radius of gyration (wild-type 6.184 ± 0.109 nm; L321F 6.025 ± 0.335 nm; G402E 5.523 ± 0.093 nm; M711I 5.557 ± 0.097 nm). **(D)** Solvent-accessible surface area (wild-type 486.41 ± 4.84 nm^2^; L321F 491.24 ± 4.79 nm^2^; G402E 484.84 ± 5.03 nm^2^; M711I 492.40 ± 4.72 nm^2^). **(E)** Total CUL3:KEAP1 intra-complex H-bond count (wild-type 6.49 ± 2.17; L321F 6.08 ± 1.91; G402E 7.71 ± 1.99; M711I 8.33 ± 2.13). Thin traces are raw data; thick lines are 5 ns rolling means. Black = CUL3 wild-type; red = CUL3 L321F; blue (#3240a8) = CUL3 G402E; green (#2a9666) = CUL3 M711I.

**FIGURE 8:**
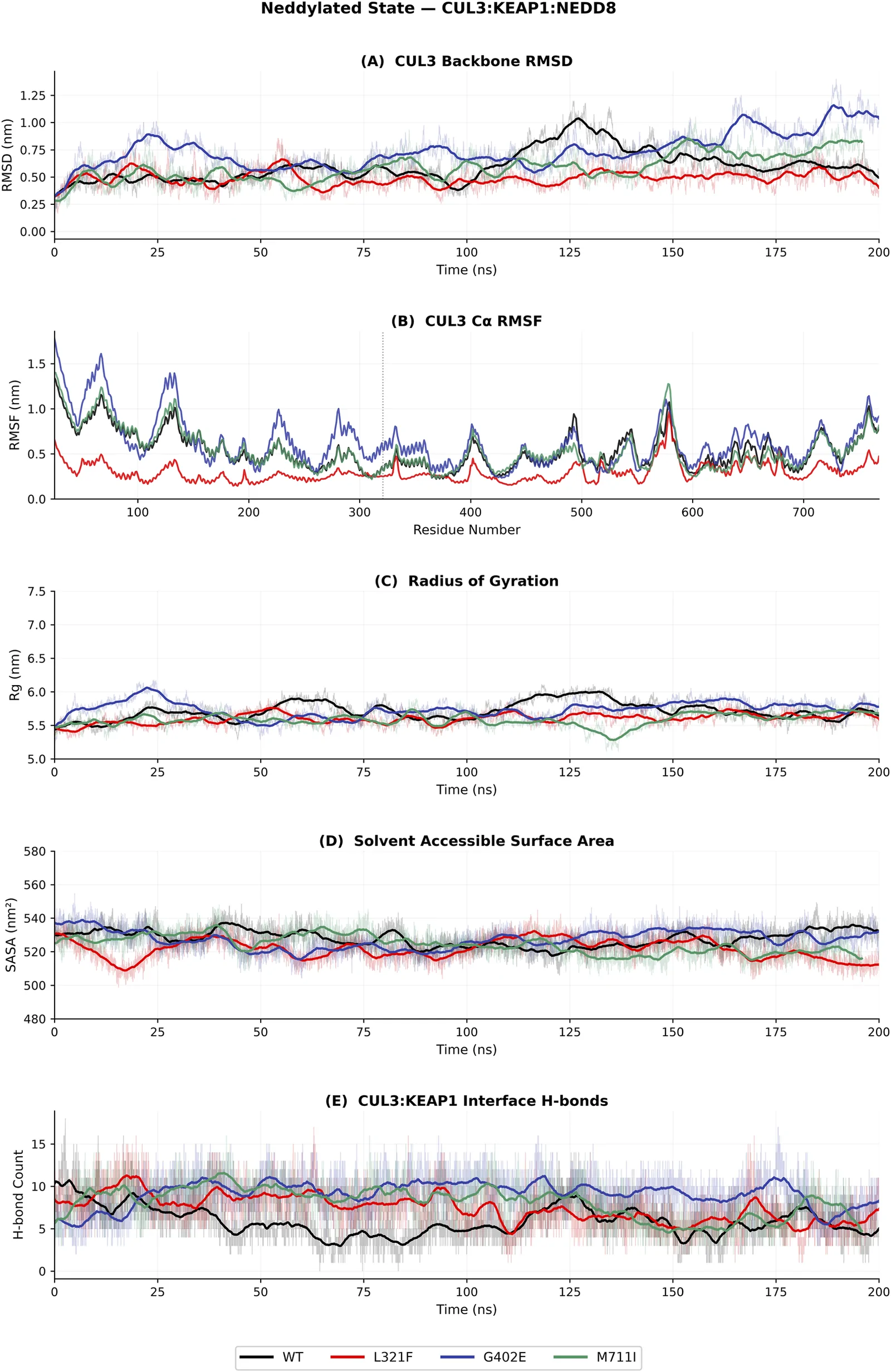
Molecular dynamics trajectory analysis neddylated state (CUL3:KEAP1:NEDD8) for four CUL3 variants. **(A)** CUL3 backbone RMSD over the production trajectory; WT + NEDD8 0.597 ± 0.155 nm; L321F + NEDD8 0.489 ± 0.091 nm; G402E + NEDD8 0.740 ± 0.174 nm; M711I + NEDD8 0.585 ± 0.143 nm. **(B)** Per-residue CUL3 Cα RMSF (WT + NEDD8 0.532 ± 0.205 nm; L321F + NEDD8 0.295 ± 0.107 nm; G402E + NEDD8 0.628 ± 0.275 nm; M711I + NEDD8 0.537 ± 0.225 nm); dotted vertical line indicates residue 321. **(C)** Radius of gyration (WT + NEDD8 5.714 ± 0.151 nm; L321F + NEDD8 5.603 ± 0.098 nm; G402E + NEDD8 5.736 ± 0.135 nm; M711I + NEDD8 5.584 ± 0.113 nm). **(D)** Solvent-accessible surface area (WT + NEDD8 527.88 ± 6.41 nm^2^; L321F + NEDD8 521.66 ± 6.95 nm^2^; G402E + NEDD8 527.38 ± 6.82 nm^2^; M711I + NEDD8 524.44 ± 6.91 nm^2^). **(E)** CUL3:KEAP1 interface H-bond count (WT + NEDD8 5.88 ± 2.30; L321F + NEDD8 7.44 ± 2.30; G402E + NEDD8 9.13 ± 2.20; M711I + NEDD8 8.14 ± 2.38). Thin traces are raw data; thick lines are 5 ns rolling means. The WT + NEDD8 backbone-RMSD excursion at ∼100–150 ns (panel A) is transient (20 ns block-averaged peak 1.20 nm at ∼126 ns, relaxing to ∼0.60 nm), whereas G402E + NEDD8 shows a progressive backbone-RMSD drift across the trajectory (block means rising 0.58→1.03 nm). All systems were simulated to 200 ns except M711I + NEDD8 (196 ns). Black = WT + NEDD8; red = L321F + NEDD8; blue (#3240a8) = G402E + NEDD8; green (#2a9666) = M711I + NEDD8.

Per-residue RMSF analysis reinforced this interpretation. In the inactive state, the wild-type complex showed uniformly higher Cα flexibility across the CUL3 scaffold relative to L321F (mean RMSF: WT 0.727 ± 0.375 nm; L321F 0.436 ± 0.204 nm; Figure 7B). In the neddylated state, this divergence was preserved and extended across the CUL3 scaffold (WT + NEDD8: 0.532 ± 0.205 nm; L321F + NEDD8: 0.295 ± 0.107 nm; Figure 8B), indicating a concerted reduction in per-residue dynamics in the mutant neddylated complex. The radius of gyration and solvent-accessible surface area were correspondingly reduced in the L321F neddylated system (Rg: 5.603 ± 0.098 nm vs. 5.714 ± 0.151 nm; SASA: 521.66 ± 6.95 nm^2^ vs. 527.88 ± 6.41 nm^2^; Figures 8C,D), consistent with a more compact interface geometry in the mutant. Hydrogen bond analysis at the CUL3:KEAP1 interface (Chain A–B) revealed elevated contact counts in L321F + NEDD8 (7.44 ± 2.30) relative to WT + NEDD8 (5.88 ± 2.30; Figure 8E), while chain-resolved CUL3:NEDD8 interface contacts (Chain A–C) were substantially higher in the mutant neddylated system (6.63 ± 2.81 vs. 3.35 ± 1.84). Given that residue 321 is located approximately 390 residues from the K712 neddylation site, this elevation in CUL3:NEDD8 contacts points to long-range allosteric propagation through the Cullin scaffold rather than a local structural perturbation. The neddylated mutant systems were likewise characterised: G402E + NEDD8 showed elevated per-residue flexibility (RMSF 0.628 ± 0.275 nm) consistent with its higher backbone RMSD, while M711I + NEDD8 was intermediate (0.537 ± 0.225 nm; Figure 8B). Radius of gyration and SASA for both new neddylated mutants fell within the wild-type range (G402E + NEDD8 Rg 5.736 ± 0.135 nm, SASA 527.38 ± 6.82 nm^2^; M711I + NEDD8 Rg 5.584 ± 0.113 nm, SASA 524.44 ± 6.91 nm^2^; Figures 8C,D), and CUL3:KEAP1 interface H-bond counts were elevated in both (G402E + NEDD8 9.13 ± 2.20; M711I + NEDD8 8.14 ± 2.38; Figure 8E). Thus, among the four neddylated variants, L321F is distinguished by concerted rigidification, whereas G402E is distinguished by a progressive backbone destabilisation, indicating that different CUL3 missense substitutions perturb the neddylated scaffold in mechanistically distinct ways.

### 3.13 Conformational landscape analysis

Principal component analysis of CUL3 Cα coordinates revealed qualitatively distinct conformational dynamics across all eight simulated systems (Figure 9). In the inactive state, PC1 and PC2 together captured 49.9%, 41.5%, 58.3% and 50.3% of total conformational variance for wild-type, L321F, G402E and M711I, respectively; wild-type sampled a broader amplitude (PC1 range 33.8 nm; λ_1_ = 19.46 nm^2^) than L321F (25.0 nm; λ_1_ = 16.80 nm^2^), whereas G402E showed the largest collective motion of the inactive panel (PC1 range 37.6 nm; λ_1_ = 40.17 nm^2^), indicating that the L321F substitution alone restricts conformational sampling even in the absence of neddylation. In the neddylated state this divergence was amplified: WT + NEDD8 expanded markedly (PC1 variance 43.1%; λ_1_ = 45.05 nm^2^; PC1 range 43.3 nm) and G402E + NEDD8 expanded further still (45.4%; λ_1_ = 54.08 nm^2^; PC1 range 43.4 nm), while M711I + NEDD8 occupied an intermediate landscape (38.7%; λ_1_ = 37.31 nm^2^) and L321F + NEDD8 alone contracted (24.9%; λ_1_ = 18.30 nm^2^; PC1 range 26.4 nm). Across both states the free-energy landscapes of wild-type, G402E and M711I are broad and multi-welled, whereas L321F remains confined to a single narrow basin (Figures 9B,J), indicating that neddylation broadly expands the conformational space of the wild-type and the two recurrent-mutant scaffolds but fails to do so in L321F, where the scaffold appears pre-constrained to an alternative preferred conformation.

**FIGURE 9:**
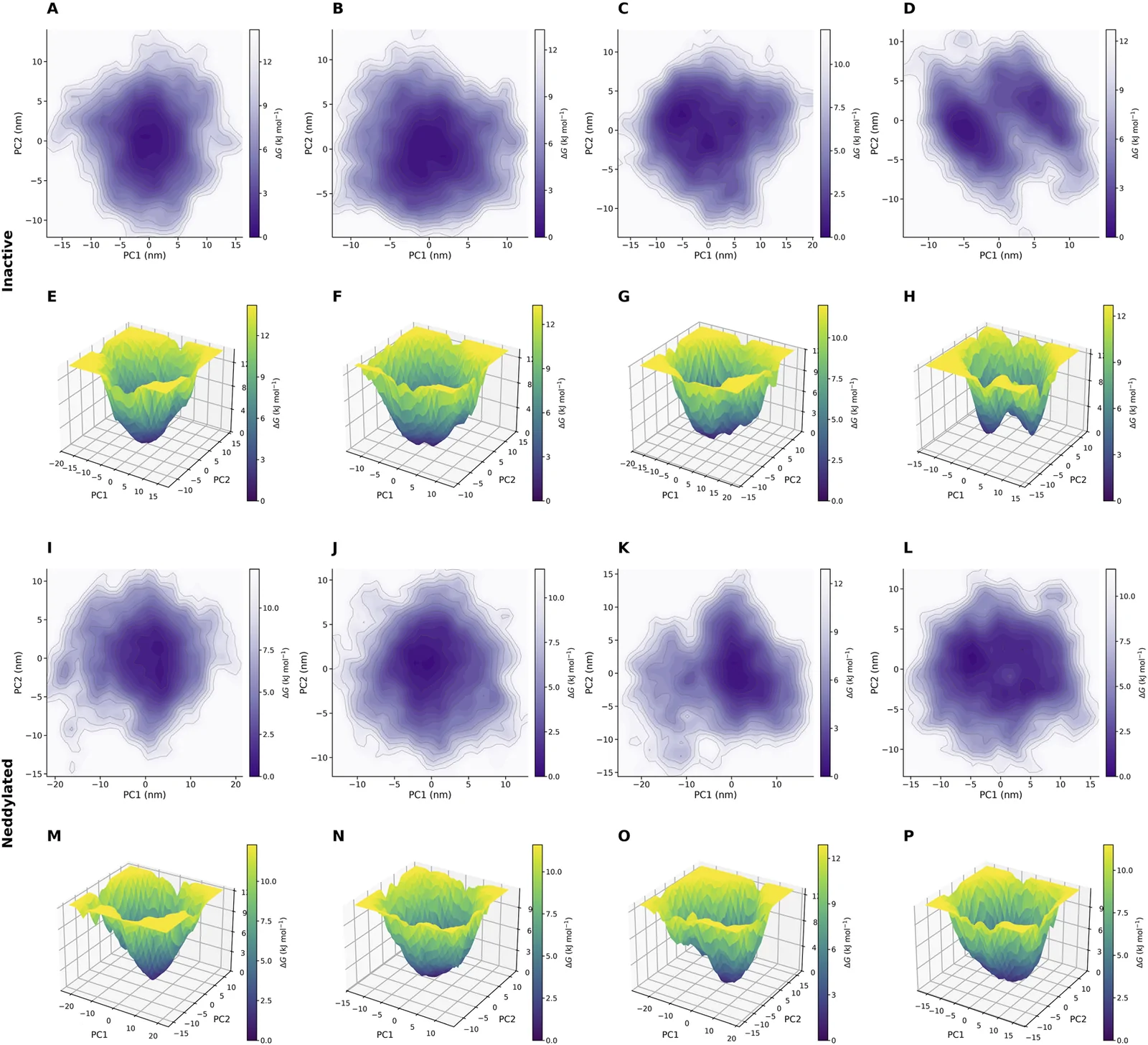
Free energy landscapes of the CUL3:KEAP1 (:NEDD8) complex across four CUL3 variants in both functional states. Free energy landscapes (FEL) constructed from the PC1–PC2 projection of CUL3 Cα coordinates for wild-type, L321F, G402E and M711I in the inactive (top two rows) and neddylated (bottom two rows) states. For each system the 2D contour (panels **(A–D)** and **(I–L)** and the corresponding 3D surface (panels **(E–H)** and **(M–P)** are shown. The colour scale represents ΔG (kJ mol^-1^) computed as −RT ln(P) at 310 K; dark = lowest-energy (most populated) basin. PC1+PC2 cumulative variance: inactive WT 49.9%, L321F 41.5%, G402E 58.3%, M711I 50.3%; neddylated WT 58.4%, L321F 45.6%, G402E 64.0%, M711I 56.0%. Wild-type, G402E and M711I sample broad, multi-welled basins that widen upon neddylation (λ_1_ rising from 19.46, 40.17 and 31.10 nm^2^ to 45.05, 54.08 and 37.31 nm^2^, respectively), whereas L321F remains confined to a single narrow basin in both states (λ_1_ 16.80 → 18.30 nm^2^), consistent with allosteric rigidification to an alternative preferred conformation.

Free energy landscape analysis directly visualised this divergence. The WT + NEDD8 landscape displayed two distinct low-energy basins (≤1 kT) with eight local minima (≤5 kJ mol^-1^), reflecting broad conformational sampling across multiple accessible substates. In contrast, the L321F + NEDD8 landscape converged to a single confined basin with four local minima, with its global minimum shifted to (PC1 = −0.20 nm, PC2 = +0.28 nm) relative to WT + NEDD8 (PC1 = +2.70 nm, PC2 = −4.30 nm). The reduced conformational breadth and altered basin position in L321F + NEDD8 are consistent with allosteric rigidification restricting the complex to an alternative preferred geometry, likely impairing the productive conformational transitions required for CRL3-mediated NRF2 ubiquitination.

## 4 Conclusion

This study integrates multi-cohort genomic analysis, transcriptomic profiling, and all-atom molecular dynamics simulation to characterise CUL3 as a context-dependent modifier of NRF2 pathway regulation in EGFR-driven non-small cell lung cancer. The convergent findings across three analytical phases support a mechanistic model in which CUL3 missense variants, exemplified by L321F, operate through allosteric rigidification of the neddylation-active CRL3 complex. Genomic analysis established that, CUL3-mutation enrichment is driven by TP53 alteration rather than by EGFR–TP53 synergy, the EGFR × TP53 interaction being non-significant and is observed within the EGFR-oncogenic disease background, robust to sequential NRF2-axis confounder exclusion and preserved across six sensitivity scenarios. Transcriptomic profiling confirmed elevated NRF2 pathway activation as the functional consequence, alongside proliferative signalling, immune microenvironment remodelling, and altered drug metabolism gene expression consistent with NRF2-driven reprogramming. Molecular dynamics simulation revealed that the L321F substitution drives the neddylated complex into a conformationally restricted state with a shifted energy minimum and reduced conformational sampling amplitude, indicating impaired catalytic flexibility despite preserved complex integrity. Because each system was simulated as a single trajectory, the variant-specific conformational differences are interpreted comparatively across the four CUL3 backgrounds and would require independent replicates for formal statistical confidence. The neddylation pathway co-expression data, together with the structural findings, support a model in which CUL3 mutations represent a non-canonical but functionally relevant route to NRF2 axis dysregulation in this subgroup. As an integrative computational study, these convergent genomic, transcriptomic, and structural results are advanced as hypotheses for experimental testing through NRF2 reporter, CRL3:KEAP1 co-immunoprecipitation, and *in-vitro* neddylation-kinetics assays in CUL3-variant models. The identification of CUL3 variation as a TP53-associated, NRF2-activating event would nominate CUL3-mutant, TP53-altered adenocarcinoma as a candidate context for NRF2-pathway or neddylation-directed intervention and for NRF2-based risk stratification, a direction for future translational investigation.

## Data Availability

The original contributions presented in the study are included in the article/Supplementary Material, further inquiries can be directed to the corresponding author.

## Supplementary material

SUPPLEMENTARY FIGURE S1 Somatic mutation landscape and pairwise co-occurrence patterns in the EGFR-oncogenic cohort (n = 10,228). **(A)** OncoPrint of five canonical genes across Triple-Positive (n = 61), EGFR + TP53 Niche (n = 5,666), and EGFR-Only (n = 4,501) groups; samples sorted by TMB descending within each group. **(B)** Pairwise co-occurrence heatmap; tile colour = −log_10_(p); co-mutation counts shown inside tiles; CUL3–TP53 highlighted (n = 76, p = 4.1 × 10^−5^); all 10 pairs significant (p < 0.05).

SUPPLEMENTARY FIGURE S2 NRF2 pathway activation significance and tumour mutational burden distribution across mutation-defined groups. **(A)** Radar plot of −log_10_(p) for NRF2 activation across three canonical gene signatures; dashed circle = p = 0.05; held-out validation split (n = 36, exact Wilcoxon; wine) and full cohort (n = 120; orange) results shown. **(B)** Per-sample TMB waterfall (n = 1,996) sorted by descending TMB and coloured by group; dashed line = TMB-High threshold (10 mut/Mb).
