# Supplementary material for: CUL3-mediated NRF2 dysregulation in TP53-driven non-small cell lung cancer: neddylation-associated structural perturbations
Source: Front Bioinform. 2026 Oct 30;6:1918128. doi: 10.3389/fbinf.2026.1918128 (PMC13759385; doi:10.3389/fbinf.2026.1918128)
Supplement: Supplementary file 1 [file Table1.docx]

**Supplementary Table 1. Sequential NRF2-axis exclusion scenarios and canonical Triple-Positive cohort definition.**

Six sequential sensitivity scenarios applied to the pre-sensitivity Triple-Positive cohort (N = 76). The canonical cohort (highlighted, N = 61) is defined after hypermutator and NFE2L2-mutant exclusion. CUL3 enrichment in the EGFR/TP53 background was preserved across all six scenarios.

| **Exclusion Scenario** | **N Excluded (cumulative)** | **N Triple-Positive Retained** | **KEAP1 Excluded** | **STK11 Excluded** | **NFE2L2 Excluded** |
| --- | --- | --- | --- | --- | --- |
| Base (Any EGFR-oncogenic) | 0 | 76 | No | No | No |
| Sensitivity 1: +Hypermutator exclusion (TMB ≥100 mut/Mb) | 8 | 68 | No | No | No |
| Sensitivity 2: +NFE2L2 exclusion [CANONICAL] | 15 | 61 | No | No | Yes |
| Sensitivity 3: +KEAP1 exclusion | 28 | 48 | Yes | No | Yes |
| Sensitivity 4: +STK11 exclusion | 24 | 52 | No | Yes | Yes |
| Sensitivity 5: Combined KEAP1 OR STK11 (strictest) | 34 | 42 | Yes | Yes | Yes |

**Note:** Cumulative exclusion count is relative to Base N = 76. Hypermutator threshold: TMB ≥ 100 non-synonymous mutations/Mb.
