# Supplementary material for: CUL3-mediated NRF2 dysregulation in TP53-driven non-small cell lung cancer: neddylation-associated structural perturbations
Source: Front Bioinform. 2026 Oct 30;6:1918128. doi: 10.3389/fbinf.2026.1918128 (PMC13759385; doi:10.3389/fbinf.2026.1918128)
Supplement: Supplementary file 2 [file Table2.docx]

**Supplementary Table 2. Complete imSig ssGSEA immune microenvironment panel - all 10 canonical categories.**

ssGSEA scores computed using the canonical imSig panel (Nirmal et al. 2018; CRAN imsig v1.1.3) across 120 TCGA LUAD/LUSC patients. Wilcoxon rank-sum with Benjamini-Hochberg FDR. Sorted by FDR ascending. Proliferation (green) is the only FDR-significant finding; Interferon (yellow) shows a near-significant directional trend. Both are reported in main text.

| **imSig Category** | **Mean ssGSEA (WT, n=89)** | **Mean ssGSEA (CUL3-Mut, n=31)** | **Effect (Δ)** | **P-Value (Wilcoxon)** | **FDR** | **Direction / Note** |
| --- | --- | --- | --- | --- | --- | --- |
| Proliferation | 0.089 | 0.230 | +0.141 | 4.90 × 10⁻⁷ | 4.41 × 10⁻⁶ | Mut > WT *** |
| Interferon | 0.312 | 0.269 | −0.044 | 1.82 × 10⁻² | 8.17 × 10⁻² | Mut < WT (near-significant trend) |
| Neutrophils | 0.216 | 0.199 | −0.018 | 4.72 × 10⁻² | 1.42 × 10⁻¹ | Mut < WT (ns) |
| Translation | 0.515 | 0.520 | +0.005 | 1.23 × 10⁻¹ | 2.38 × 10⁻¹ | Mut ≈ WT (ns) |
| Monocytes | 0.271 | 0.253 | −0.018 | 1.59 × 10⁻¹ | 2.38 × 10⁻¹ | Mut < WT (ns) |
| T cells | 0.045 | 0.016 | −0.029 | 1.79 × 10⁻¹ | 2.38 × 10⁻¹ | Mut < WT (ns) |
| Macrophages | 0.251 | 0.223 | −0.027 | 1.85 × 10⁻¹ | 2.38 × 10⁻¹ | Mut < WT (ns) |
| NK cells | −0.299 | −0.286 | +0.013 | 3.00 × 10⁻¹ | 3.37 × 10⁻¹ | Mut > WT (ns) |
| B cells | −0.116 | −0.138 | −0.022 | 4.57 × 10⁻¹ | 4.57 × 10⁻¹ | Mut < WT (ns) |
| Plasma cells | n/a | n/a | n/a | n/a | n/a | Insufficient gene coverageᵃ |

**Note:** *** FDR < 0.001. ns = not significant. n/a = not applicable.

Plasma cells: excluded from ssGSEA because only 4/14 imSig Plasma Cell genes recovered in TCGA RSEM matrix (below GSVA minSize = 5 threshold). Reflects inconsistent annotation of immunoglobulin locus identifiers in bulk RNA-seq pipelines.
