# Supplementary material for: CUL3-mediated NRF2 dysregulation in TP53-driven non-small cell lung cancer: neddylation-associated structural perturbations
Source: Front Bioinform. 2026 Oct 30;6:1918128. doi: 10.3389/fbinf.2026.1918128 (PMC13759385; doi:10.3389/fbinf.2026.1918128)
Supplement: Supplementary file 3 [file Table3.docx]

**Supplementary Table 3. Complete NRF2-regulated drug metabolism gene panel -Wilcoxon rank-sum results (n = 18 genes).**

All 18 NRF2-regulated drug metabolism genes tested by Wilcoxon rank-sum comparing CUL3-mutant (n = 31, including 2 Triple-Positive) versus CUL3 wild-type (n = 89) TCGA LUAD/LUSC patients. Z-score normalised RSEM expression values. Sorted by FDR ascending. Genes reaching FDR < 0.05 (highlighted) are reported in main text Table 3.

| **Gene** | **Phase / Enzyme Class** | **Median Z (WT)** | **Median Z (CUL3-Mut)** | **ΔZ** | **P-Value** | **FDR** | **Significance** |
| --- | --- | --- | --- | --- | --- | --- | --- |
| *UGT1A1* | Phase II — UGT conjugation | −1.65 | −0.34 | +1.32 | 1.09 × 10⁻⁵ | 1.97 × 10⁻⁴ | *** |
| *CYP3A5* | Phase I — CYP oxidation | 0.11 | −0.78 | −0.89 | 9.98 × 10⁻⁵ | 8.98 × 10⁻⁴ | *** |
| *UGT1A9* | Phase II — UGT conjugation | −1.71 | 0.46 | +2.17 | 2.02 × 10⁻³ | 1.21 × 10⁻² | * |
| *UGT2B7* | Phase II — UGT conjugation | −0.41 | −0.94 | −0.53 | 9.19 × 10⁻³ | 4.14 × 10⁻² | * |
| *CYP1A1* | Phase I — CYP oxidation | −0.67 | −0.23 | +0.44 | 1.62 × 10⁻² | 5.85 × 10⁻² | ns |
| *UGT1A6* | Phase II — UGT conjugation | −0.29 | 0.48 | +0.77 | 8.97 × 10⁻² | 2.69 × 10⁻¹ | ns |
| *CYP2C8* | Phase I — CYP oxidation | −0.10 | −0.89 | −0.79 | 1.11 × 10⁻¹ | 2.85 × 10⁻¹ | ns |
| *ABCC2* | Phase III — ABC efflux | −0.47 | −0.42 | +0.04 | 1.29 × 10⁻¹ | 2.91 × 10⁻¹ | ns |
| *GSTP1* | Phase II — GST conjugation | −0.31 | 0.02 | +0.33 | 1.68 × 10⁻¹ | 3.36 × 10⁻¹ | ns |
| *CYP2C9* | Phase I — CYP oxidation | −0.70 | −0.87 | −0.16 | 3.70 × 10⁻¹ | 6.05 × 10⁻¹ | ns |
| *ABCC3* | Phase III — ABC efflux | 0.28 | 0.37 | +0.10 | 3.34 × 10⁻¹ | 6.02 × 10⁻¹ | ns |
| *ABCC4* | Phase III — ABC efflux | 0.15 | 0.15 | −0.003 | 7.15 × 10⁻¹ | 7.57 × 10⁻¹ | ns |
| *ABCG2* | Phase III — ABC efflux | 0.18 | −0.20 | −0.38 | 5.13 × 10⁻¹ | 7.57 × 10⁻¹ | ns |
| *GSTM1* | Phase II — GST conjugation | −0.77 | −0.66 | +0.12 | 8.03 × 10⁻¹ | 8.03 × 10⁻¹ | ns |
| *CYP1B1* | Phase I — CYP oxidation | −0.02 | 0.02 | +0.04 | 6.84 × 10⁻¹ | 7.57 × 10⁻¹ | ns |
| *GSTA1* | Phase II — GST conjugation | 0.04 | 0.08 | +0.04 | 6.88 × 10⁻¹ | 7.57 × 10⁻¹ | ns |
| *CYP3A4* | Phase I — CYP oxidation | −0.91 | −1.06 | −0.14 | 7.15 × 10⁻¹ | 7.57 × 10⁻¹ | ns |
| *GSTM3* | Phase II — GST conjugation | 0.12 | −0.03 | −0.15 | 6.62 × 10⁻¹ | 7.57 × 10⁻¹ | ns |

**Note:** ΔZ = difference in group medians (CUL3-mutant − WT). *** FDR < 0.001; * FDR < 0.05; ns = not significant (Benjamini-Hochberg).
